# New Proposal of Setal Homology in Schizomida and Revision of *Surazomus* (Hubbardiidae) from Ecuador

**DOI:** 10.1371/journal.pone.0147012

**Published:** 2016-02-10

**Authors:** Osvaldo Villarreal Manzanilla, Gustavo Silva de Miranda, Alessandro Ponce de Leão Giupponi

**Affiliations:** 1 Departamento de Invertebrados, Pós-Graduação em Zoologia, Museu Nacional da Universidade Federal do Rio de Janeiro, Rio de Janeiro, RJ, Brazil; 2 Center for Macroecology, Evolution and Climate, Natural History Museum of Denmark (Zoological Museum), University of Copenhagen, Copenhagen, Denmark; 3 Laboratório de Referência Nacional em Vetores das Riquetsioses, LIRN-FIOCRUZ, Rio de Janeiro, RJ, Brazil; Australian Museum, AUSTRALIA

## Abstract

The homology of three somatic systems in Schizomida is studied yielding the following results: (1) proposal of homology and chaetotaxy of abdominal setae in *Surazomus*; (2) revision of the cheliceral chaetotaxy in Schizomida, with suggestion of new homology scheme between Hubbardiidae and Protoschizomidae, description of a new group of setae in Hubbardiinae (G7), and division of setae group 5 in two subgroups, G5A and G5B; (3) proposal of segmental homology between trimerous and tetramerous female flagellum in Hubbardiinae with association of segment III of tri-segmented species to segments III + IV of tetra-segmented species. Considerations about the dorsal microsetae on the male flagellum are made. The genus *Surazomus* in Ecuador is revised. The sympatric species *Surazomus palenque* **sp. nov.** and *S*. *kitu* **sp. nov.** (Ecuador, Pichincha) are described and illustrated. The female of *S*. *cuenca* (Rowland and Reddell, 1979) is described, with two new distributional records for the species. *Surazomus cumbalensis* (Kraus, 1957) is recorded for the first time from Ecuador (Pichincha).

## Introduction

South American schizomids are poorly known regarding their taxonomy, evolution and ecology [1]. From Ecuador, only two species have been recorded until now: *Surazomus cuenca* (Rowland and Reddell, 1979) from Azuay province, and *Tayos ashmolei* (Reddell and Cokendolpher, 1984), the only known *Tayos* Reddell and Cokendolpher, 1995 species, from Morona-Santiago province [2]. The low number of short-tailed whipscorpion species from this country is probably due to a lack of collecting effort, but recent studies of Ecuadorian fauna of Schizomida allowed us to obtain interesting results, published in this paper.

The genus *Surazomus* Reddell and Cokendolpher, 1995 has 20 described species, which are found in Bolivia (one species), Brazil (seven), Colombia (three), Costa Rica (seven), Ecuador (one) and Peru (one) [3, 4]. Furthermore, at least 10 new undescribed species are known from Colombia, Costa Rica, Ecuador and Panama [4–6]. This genus is defined by having the spermathecae with one pair of two digit-like lobes, usually with a distal expansion, absence of chitinized arc and gonopod, and flagellum of the male dorsoventrally flattened, generally with dorsal sculpturing and lateral lobules well developed. Pedipalps are sexually dimorphic, and ornamentation of pedipalps and shape of the flagellum may vary among different species groups. The chelicera is also an important source of characters to define species, mainly the number of teeth in the movable and fixed finger, the presence/absence of a guard tooth, and the number of setae. However, for some reason, the cheliceral setae have been neglected in the taxonomy of the order.

Hansen and Sörensen [7] were the first to name some of the schizomid cheliceral setae, as the remarkable "blood-hairs", and Lawrence [8] was the first to establish a chaetotaxy system, defining groups of setae according to their morphology, position and number. Lawrence [8] proposed six groups in the mesal side of the chelicerae (groups 1 to 6, or G1-G6) and this chaetotaxy system was used by several authors thereafter [9–13], but recent papers just count the number of setae per group (e. g. [14–18]),ignoring their relative position, shape and size, as originally proposed. Besides that, most of the descriptions of chelicerae deal with Hubbardiidae species, including the original proposal of Lawrence [8], and contributions to the understanding of Protoschizomidae chelicerae are rare. Cokendolpher and Reddell [13] were the first to use Lawrence chaetotaxy in Protoschizomidae, counting five groups in the chelicerae of *Agastoschizomus huitzmolotitlensis* Rowland, 1975 (they considered G5 absent in this family) [19]. Another work that recorded the cheliceral setae in Protoschizomidae was Monjaraz-Ruedas [16], who counted six setal groups (the same number as Hubbardiidae) in a species of the genus *Protoschizomus* Rowland, 1975. However, the homology of these setae between the living families, and the variation of the setae among Schizomida genera have never been analyzed and discussed. In this paper, a review of the current proposal of the cheliceral chaetotaxy is presented with a new hypothesis of homologies between the living families. Moreover, two new groups of setae on the chelicerae are proposed.

The chaetotaxy of Schizomida has been described for some of the body structures, such as the chelicerae (as mentioned above) and the flagellum [13, 20]. However, the abdominal setae have been neglected as a source of characters, being investigated in only a few taxonomic works [21, 22]. As a consequence, no homology or nomenclature has been proposed for those setae. A careful observation of the abdomen shows that the tergites have two types of setae that can be identified by their size: the macrosetae and the microsetae. The former have been described as a pair of setae in the medial region of the tergites, while the latter have been reported as an accessory series of setae, medial to the macrosetae. The microsetae on the first two abdominal tergites were also identified as two anterior longitudinal rows of setae [20]. To standardize the names and make the first step towards an evolutionary understanding of those setae, we propose a homology hypothesis and a nomenclature for schizomid abdominal setae (the names are similar to those of the flagellum as the setae are named after the position in the tergites and sternites, but the setae of these structures are not homologues; see topic ‘Nomenclature of structures and conventions’ for more details).

Additionally, we describe two new species of *Surazomus* from Ecuador (province of Pichincha) and the hitherto unknown female of *S*. *cuenca*. *Surazomus cumbalensis* (Kraus, 1957) is newly recorded from that country.

## Material and Methods

### Material examined and laboratory procedures

#### Ethics statement

Part of the material here studied was collected in Ecuador under the permit 004–14 IC-FAU-DNB/MA (Ministerio del Ambiente, Ecuador). The areas sampled were privately owned. The species collected are not protected and no protected species of other animal group were taken from nature. The animals were immediately preserved in 75% alcohol for subsequent laboratory studies.

The material of *Surazomus kitu* **sp.nov.** and *S*. *palenque* **sp. nov.** (holotypes and paratypes) are permanently deposited at Museo de Zoologia/Pontificia Universidad Católica de Quito (QCAZ) and are on loan to Museu Nacional/Universidade Federal de Rio de Janeiro (MNRJ). Paratypes of *S*. *palenque* **sp. nov.** were donated to MNRJ and are permanently deposited there. Non-type material of *S*. *cumbalensis* are permanently deposited at Museu Nacional/Universidade Federal de Rio de Janeiro (MNRJ) and Museo de Zoologia/Pontificia Universidad Católica de Quito (QCAZ). Non-type material of *S*. *cuenca* are deposited at MNRJ. The holotype of *S*. *cuenca* is permanently deposited at the American Museum of Natural History, New York (AMNH). Additional material examined were *S*. *chavin* Pinto-da-Rocha, 1996 (paratypes at the AMNH, no number; non-type material at the Museu de Zoologia/Universidade de São Paulo, MZUSP, no number, [23]), *S*. *boliviensis* Cokendolpher and Reddel, 2000 (holotype at the AMNH, no number, [24]), and *S*. *pallipatellatus* (Rowland and Reddel, 1979) (holotype at the AMNH, no number [25]).

#### Drawings and photographs

The drawings of spermathecae were made using Inkscape [26] software and are based on photographs taken on a Nikon microscope with a Sony DSC V1 digital camera. Spermathecae were prepared by submersion in lactophenol for no less than two hours, and then placed in a micro-vial with the dissected female. The chelicerae and flagellum were prepared for Scanning Election Microscopy (SEM) with triple ultrasonication in water and detergent, critical point dried, gold coated, and examined with a JEOL JSM-6390LV at the Center for Scanning Electron Microscopy of Museu Nacional/Universidade Federal do Rio de Janeiro and SEM Platform Rudolf Barth at Instituto Oswaldo Cruz—Fundação Oswaldo Cruz (IOC-FIOCRUZ).

### Nomenclature of structures and conventions

The nomenclature of pedipalps, legs and spermathecae follows Reddell and Cokendolpher [3]; the nomenclature for flagellum setation follows Harvey [20], modified by Cokendolpher and Reddell [13], Villarreal, Armas and García [18] and Moreno-González, Delgado-Santa and Armas [27]; the nomenclature of cheliceral setae is based on Lawrence [8]; the chelicerae of Protoschizomidae were analyzed based on figures in the literature [13, 16, 28], and some unpublished SEM images of *Agastoschizomus lucifer* Rowland, 1971 [29], accessible in Morphbank [30]. Tables 1 and 2 summarize which species had specimens analyzed under the microscope and which had details extracted from the literature. Descriptions follow Moreno-González and Villarreal [14]. The spots on the abdominal sternites, called respiratory spiracles by Teruel [21], Armas and Teruel [31], Villarreal, Giupponi and Tourinho [32], Villarreal and García [15] and Moreno-González and Villarreal [14], and branchial spots by Montaño-Moreno and Francke [33], are here called abdominal apodemes

**Table 1:** List of sources (vouchers) used for comparative discussion.

Taxa	Vouchers*
*Surazomus boliviensis* Cokendolpher and Reddel, 2000	1 male (AMNH)
*S*. *chavin* Pinto-da-Rocha, 1996	1 male (AMNH), 1 male (MZUSP)
*S*. *cuenca* (Rowland & Reddell, 1979)	1 male (AMNH)
*S*. *cumbalensis* (Kraus, 1957)	1 male, 1 female (MNRJ 04264)
*S*. *kitu* sp. nov.	1 male (QCAZ)
*S*. *palenque* sp. nov.	1 male (QCAZ)
*S*. *pallipatellatus* (Rowland and Reddel, 1979)	1 male (AMNH)
*Wayuuzomus gonzalezspongai* Armas & Colmenares-García, 2006	1 male (MZSP 52001)

*For details on locality, coordinates, date of capture and collector, see the topic ‘Studied material’ or the original description of the known species.

**Table 2:** List of sources (from literature) used for comparative discussion.

Taxa	Reference
*Agastoschizomus lucifer* Rowland, 1971	Morphbank [30]
*Calima* Moreno-González and Villarreal, 2012	Moreno-González and Villarreal [14]: Figures 17–19, 35–37
*Draculoides brooksi* Harvey, 2001	Harvey [34]: Figure 11
*Heterocubazomus sierramaestrae* Teruel, 2007	Teruel [21]: Figure 1d
*Mayazomus* Reddell and Cokendolpher, 1995	Monjaraz-Ruedas and Francke [35]: Figures 1–3, 33–35
*Megaschizomus mossambicus* (Lawrence, 1958)	Reddell and Cokendolpher [3]: Figure 14
*Paradraculoides kryptus* Harvey, Berry, Edward and Humphreys, 2008	Harvey, Berry, Edward and Humphreys [22]: Figure 12
*Piaroa* Villarreal, Giupponi and Tourinho, 2008	Villarreal and García [15]: Figure 10
*Piaroa guipongai* Villarreal and García, 2012	Villarreal, Armas and García [18]: Figure 26
*P*. *pioi* Villarreal, Armas and García, 2014	Villarreal, Armas and García [18]: Figures 15–21, 37
*Protoschizomus franckei* Monjaraz-Ruedas, 2013	Monjaraz-Ruedas [16]: Figure 10
*Stenochrus mexicanus* (Rowland, 1971)	Rowland and Reddell [36]: Figures1–3, 18–19, 32–34, 54–57
*Surazomus arboreus* Cokendolpher and Reddell, 2000	Cokendolpher and Reddell [24]: Figures 10–15
*S*. *boliviensis* Cokendolpher and Reddell, 2000	Cokendolpher and Reddell [24]: Figure 16
*S*. *brasiliensis* (Kraus, in Kraus and Beck)	Rowland and Reddell [25]:Figure 39; Cokendolpher and Reddell [24]: Figures 17, 18
*S*. *cuenca* (Rowland and Reddell, 1979)	Rowland and Reddell [25]: Figure 40
*S*. *manaus* Cokendolpher and Reddell, 2000	Cokendolpher and Reddell [24]: Figures 19–25
*S*. *mirim* Cokendolpher and Reddell, 2000	Cokendolpher and Reddell [24]: Figures 26–30
*S*. *paitit* Bonaldo and Pinto-da-Rocha, 2007	Bonaldo and Pinto-da-Rocha [11]: Figures 1–5
*S*. *rodriguesi* Cokendolpher and Reddell, 2000	Cokendolpher and Reddell [24]: Figures 31–34
*S*. *uarini* Santos and Pinto-da-Rocha, 2009	Santos and Pinto-da-Rocha [37]: Figures 1–14

**Fig 1:**
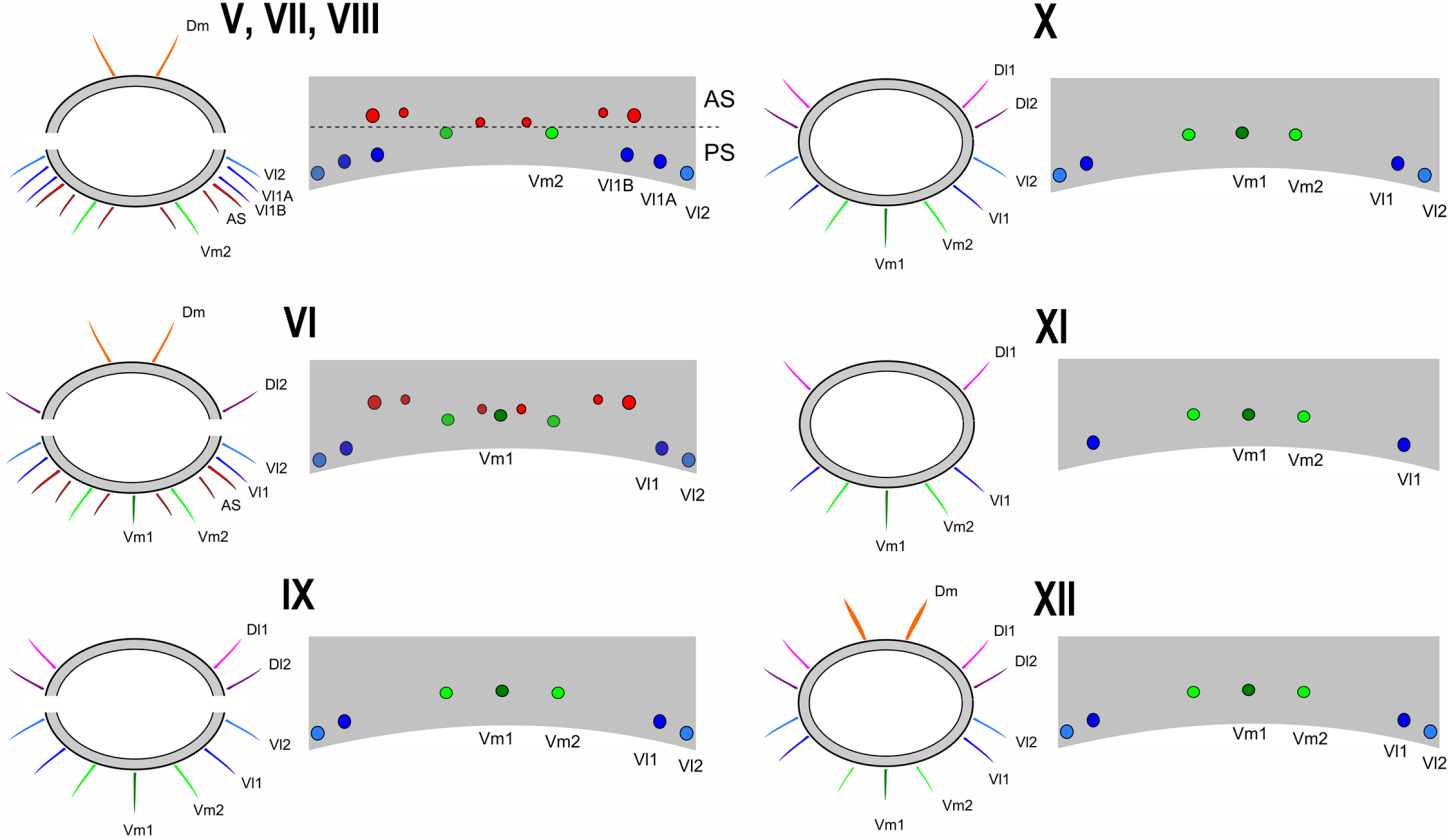
Abdominal chaetotaxy in a generalized Schizomida. The Roman numbers refer to the abdominal segment. *AS*: Anterior Series; *Dl1*: Dorso-lateral 1; *Dl2*: Dorso-lateral 2; *Dm*: Dorso-medial; *Vl1*: Ventro-lateral 1; *Vl2*: Ventro-lateral 2; *Vm1*: Ventro-medial 1; *Vm2*: Ventro-medial 2. The names refer to their position over the abdomen and are similar to that of the flagellum, but the setae of these structures are not homologous.

A nomenclature for the macrosetae on abdominal tergites and sternites is proposed (Fig 1). The names of the setae refer to their position in the plaque, and, although similar to the codes used for setae on the flagellum, these setae are not homologous. Dorso-medial (*Dm*) is the name of the pair of setae close to the centre of the tergite; Dorso-lateral 1 (*Dl1*) is the pair lateral to *Dm*; Ventro-medial 1 (*Vm1*) is the single seta in the middle of the sternite; Ventro-medial 2 (*Vm2*) is the pair of setae outside *Vm1*; Ventro-lateral 1 (*Vl1*) is the pair of setae after *Vm2*; Ventro-lateral 2 (*Vl2*) is the last lateral pair of setae in the ventral plaque.

**Fig 2:**
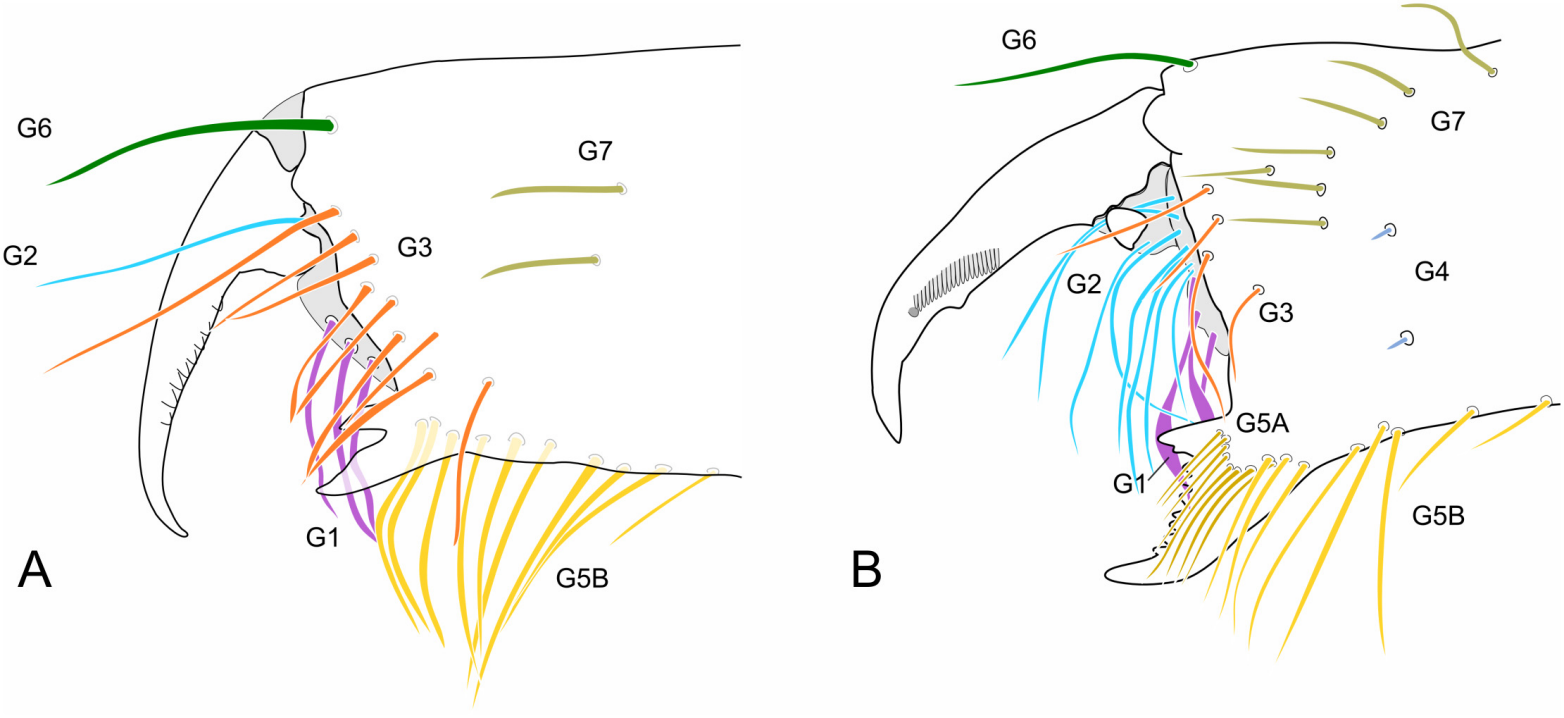
Chaetotaxy of mesal side of the chelicerae. (A) Protoschizomidae: *Protoschizomus franckei* (based in Monjaraz-Ruedas [16]: Figure 10). (B) Hubbardiidae: *Wuayuuzomus gonzalezspongai* (MZSP 52001).

A new group of setae on the upper-middle part of the mesal side of the chelicerae is identified and named as G7 (Fig 2A and 2B); the group G5 is divided into two subgroups: G5A (setae on the base of the fixed finger) and G5B (setae on the ventral border of the chelicerae; Fig 2A and 2B). The cheliceral formula in the descriptions is presented in the following order: G1-G2-G3-G4-(G5A-G5B)-G6-G7.

In almost all published descriptions of Schizomida female genitalia, the name gonopod is used for the anatomical part called ‘apophysis of the *uterus externus*’ [38, 39], but, as this structure seems not to be the path by which the spermatophore passes, this anatomical structure needs a new name. In this work we follow the classical ‘gonopod’ terminology.

### Nomenclatural acts

The electronic edition of this article conforms to the requirements of the amended International Code of Zoological Nomenclature, and hence the new names contained herein are available under that Code from the electronic edition of this article. This published work and the nomenclatural acts it contains have been registered in ZooBank, the online registration system for the ICZN. The ZooBank LSIDs (Life Science Identifiers) can be resolved and the associated information viewed through any standard web browser by appending the LSID to the prefix "http://zoobank.org/". The LSID for this publication is: urn:lsid:zoobank.org:pub:2DBC8B6B-DA0D-4129-938A-163EF9FF87FF. The electronic edition of this work was published in a journal with an ISSN, and has been archived and is available from the following digital repositories: PubMed Central, LOCKSS.

## Results

### Distribution and sympatry in Surazomus

The genus *Surazomus* has mainly an Andean-Amazonian distribution with some species in Central America, and its highest diversity is in the Colombian Andes [4, 6, 37]. It is unusual to have sympatric species of *Surazomus*; until now, just two cases of sympatry were recorded: one from the Costa Rican tropical forest, *S*. *brus* Armas, Villarreal and Viquez, 2010 and *S*. *inexpectabilis* Armas, Villarreal and Viquez, 2010 [4]; and another from the Brazilian Amazon, *S*. *arboreus* Cokendolpher and Reddell, 2000, *S*. *mirim* Cokendolpher and Reddell, 2000 and *S*. *rodriguesi* Cokendolpher and Reddell, 2000 [24]. Remarkably, *S*. *kitu* **sp. nov.** and *S*. *palenque* **sp. nov.** co-occur in the same spot, in an area close to Palenque river in Pichincha, Ecuador, this being the third case of sympatric species for the genus.

### Homology of the abdominal setae

Three rows of dorsal macrosetae on the abdominal tergites were detected in *Surazomus*: *Dm*, *Dl1* and *Dl2* (Fig 1). The distribution of these setae is as follows: *Dm* present from segment V to VIII; absent in tergite IX to XI; present in tergite XII. *Dl1* present from segment IX to XII. *Dl2* present on segments VI, IX, X and XII. Some supernumerary setae can be found, but they are not present in *Surazomus*.

The ventral setae of segment IV are aligned in one (*S*. *kitu* **sp. nov.**) or two (*S*. *cumbalensis*) transversal rows. On segments V, VII and VIII a standardization of the setae group is observed with an anterior row of six setae, named here as Anterior Series (*AS*), a posterior row with six main setae (three pairs of setae), and one pair of median setae (Fig 1). Segment VI has the same setal configuration, but with one additional seta medially (between the median pair of the *AS*), which is considered to belong to the posterior row. This extra seta is here called *Vm1*, and laterally to this seta there are three pairs of setae, one Ventromesal pair (*Vm2*) and two Lateroventral pairs (*Vl1* and *Vl2*, from the midline to the external side). In some cases some additional pairs of setae are observed that cannot be homologized to any pair of other segments by their position. These setae, when present, are called supernumerary and were observed on segments V (between the pair *Vm2*), VI, IX and XII (between *Vm2* and *Vl1*). In some of the specimens was observed asymmetry for some of these pairs, implying multiplication in one of the sides.

### Taxonomic treatment

Hubbardiidae Cook

Hubbardiinae Cook

*Surazomus* Reddel and Cokendolpher

**Fig 3:**
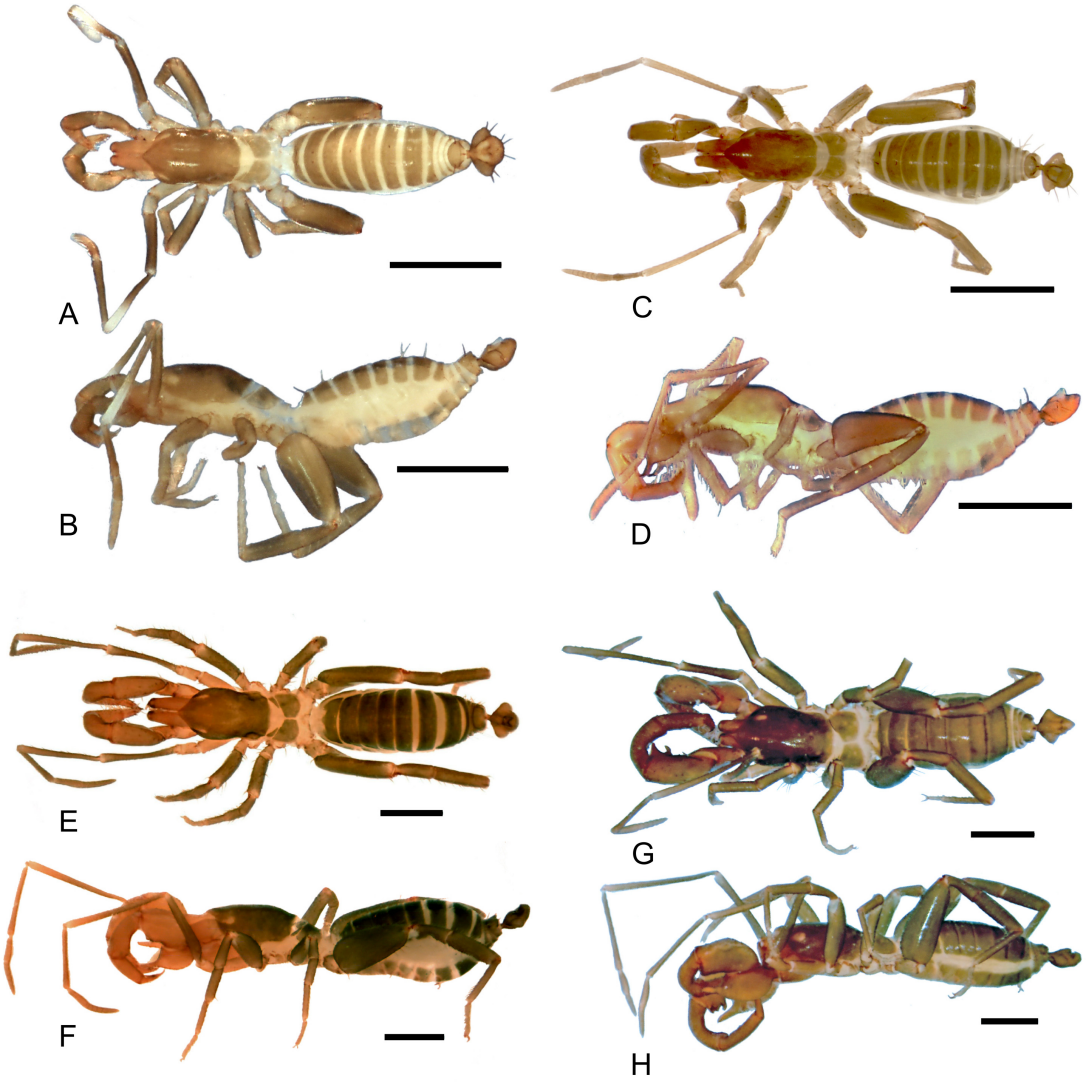
Dorsal and lateral views of the species of *Surazomus* dealt in this paper. *Surazomus kitu* **sp. n.** (male holotype), dorsal (A) and lateral (B) view; *S*. *palenque* **sp. n.** (male holotype), dorsal (C) and lateral (D) view; *S*. *cuenca* (MNRJ 04265), dorsal (E) and lateral (F) view; *S*. *cumbalensis* (MNRJ 04264), dorsal (G) and lateral (H) view. Scale bar: 1mm.

**Fig 4:**
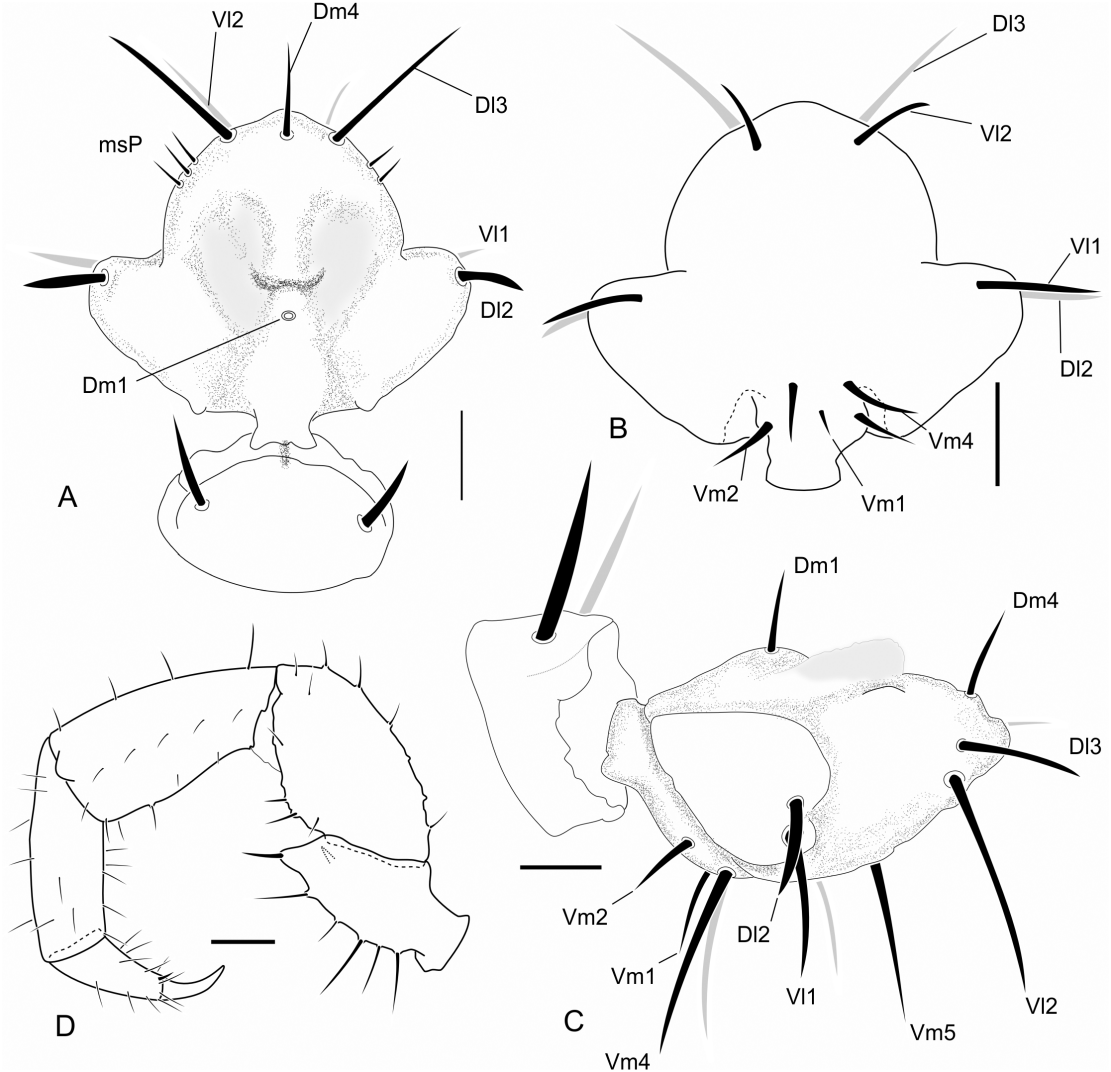
Images of details of the holotype of *Surazomus kitu* sp. n. . Dorsal (A), ventral (B), and lateral (C) view of male flagellum. Ectal view of the pedipalp (D). Scale bar: flagellum, 0.1mm; pedipalp, 0.5 mm.

***Surazomus kitu* sp. nov.**

urn:lsid:zoobank.org:act:45C4C84D-D2E7-4ACA-92DB-827F118DCD56

(Figs 3A and 3B; 4A–4D and 13; Table 3)

**Table 3:** Measurements of males of the new species here described and of females of *Surazomus cuenca*.

	*S*. *kitu* sp. nov.	*S*. *palenque* sp. nov.		*S*. *cuenca*
	Male holotype	Male holotype	Males max-min	Females max-min
**Body**				
Propeltidium: L	1.07	0.93	0.84–0.95	1.37–1.43
Propeltidium: W	0.50	0.57	0.50–0.57	0.70–0.80
Abdomen: L	1.75	1.86	1.68–1.86	2.63–2.90
Flagellum: L	0.36	0.41	0.32–0.41	0.36–0.57
Flagellum: W	0.43	0.45	0.39–0.45	0.10–0.11
Flagellum: H	0.25	0.27	0.25–030	0.10–0.11
**Pedipalp: L**				
trochanter	0.34	0.43	0.25–0.43	0.55–0.57
femur	0.34	0.59	0.36–0.95	0.57–0.67
patella	0.43	0.61	0.39–0.61	0.56–0.63
tibia	0.31	0.45	0.32–0.45	0.50–0.59
tarsus	0.20	0.20	0.16–0.30	0.23–0.27
claw	0.14	0.16	0.09–0.16	0.14–0.20
**Leg: I L**				
trochanter	0.23	0.27	0.23–0.27	0.32–0.42
femur	0.89	0.98	0.89–0.98	1.16–1.22
patella	1.00	1.18	1.09–1.18	1.32–1.39
tibia	0.70	0.77	0.66–0.77	0.97–1.00
basitarsus	0.25	0.45	0.25–0.45	0.36–0.52
telotarsus	0.45	0.30	0.30–0.45	0.35–0.55
**Leg: IV L**				
trochanter	0.23	0.30	0.23–0.30	0.20–0.33
femur	0.86	0.98	0.86–0.98	0.61–1.30
patella	0.36	0.39	0.34–0.30	0.52–0.60
tibia	0.52	0.59	0.50–0.59	0.80–0.83
basitarsus	0.48	0.57	0.45–0.57	0.73–0.77
telotarsus	0.36	0.36	0.34–0.41	0.49–0.57

**Type material—**Male holotype (QCAZ) Ecuador, Pichincha, C.C. Rio Palenque, secondary forest, 00°54´S 79°00´W, 220m, 07.i.1981, S. Sandoval.

**Diagnosis—**Small species, total length 2.82 mm (flagellum not included). Male flagellum trilobate with dorsal white spots. Absence of a ventral projection on the pedipalp femur. Presence of dorsal white spots in the flagellum projections and absence of ventral spine in the pedipalp femur. Setae *Dl*2 type B. Pedipalps unarmed, without ventral projection on the femur and patella. Patella I unpigmented, short frontal spine on the male pedipalp trochanter, lateral and distal lobes of the flagellum in dorsal view separated, in an angle of approximately 90 degrees, and relative position of *Dl*2 and *Vl*1 of the flagellum at the same horizontal level in lateral view. White area of the flagellum slightly raised. Absence of two conical tubercles in segment XII, absence of ventral projection on the femur.

**Etymology—**Name derived from the indigenous confederation inhabitant of the Ecuadorian region from where the specimens were collected; the place is known as Quitu or Kitu. It is a noun in apposition.

**Description—**Male holotype. **Coloration** (Fig 3A and 3B): general pattern light greenish-brown. Chelicerae yellowish and flagellum light brown. Pedipalps: trochanter light yellowish-brown; femur light reddish-brown; patella with distal portion unpigmented; tibia yellowish; tarsus reddish-brown. Legs: coxae I–IV, anterior and posterior sterna light yellowish; trochanters I–IV light yellowish-brown; femora I–IV dark greenish-brown; patellae I–IV light greenish-brown; tibiae II–IV light greenish-brown, except for tibia I that is reddish-brown; all tarsus light reddish-brown. All body setae light reddish-brown.

**Prosoma—**(Fig 3A) Anterior process of propeltidium with 2 setae (one behind the other) followed by 3 pairs of dorsosubmedian setae transversally oriented; eyespot suboval; metapeltidium divided. Anterior sternum with 2+10 setae and posterior sternum with 6 setae.

**Opisthosoma—**(Fig 3A and 3B) Setae: Tergite II with three pairs of anterior microsetae, with the median pair of setae is more apart from each other than the anterior and posterior. Segments II–VIII each with one pair of large *Dm* setae; segment VIII with small *Dl1*; IX without *Dm*, with pairs *Dl1* and *Dl2* present; *Vm1*, *Vm2* and pair *Vl1* present. Segment X same as IX, but without *Dl2*. Segment XI same as X, but without *Vl2*. Segment XII same as IX, with the medial *Vm1* and *Vm2* shorter than *Vl1*. Segment XII without posterodorsal process. Abdominal apodemes with coloration identical to the rest of the sternites. Sternites I–II with many scattered microsetae, III–IX each with one row of transverse microsetae.

**Flagellum—**(Fig 4A–4C) Flagellum trilobate in dorsal view; with two dorsal and curved white projections bordering the anterior margin of a depression; with two pairs of microsetae at each side of *Dm1*, the pair over a small granule in a granulose area is *Dm3*, the pair distal to *Dm3* is *Dm3B*; in the distolateral region three micro setae are present. *Dm1* located on the anterior third, on a longitudinal elevation, between the bases of the dorsal projections; *Dm4* distal, between *Dl3* in dorsal view; two microsetae between *Dl3*. *Dl2* type B located on lateral lobes, slightly posterior to *Dm1* in dorsal and lateral view; the remaining setae are type A (types of setae on flagellum are discussed below). Ventrally convex in lateral view, with two microsetae on each side of the peduncle, *Vm2* anterior to *Vm1*; *Vm1* anterior to *Vm4*; the distance between *Vm2* setae larger than that of *Vm4*; *Vm4* larger than *Vm1* and *Vm2*; *Vl1* slightly posterior and ventral to *Dl2*, located at the distal end of the lateral lobe in lateral view; *Vl2* slightly anterior to *Dl3* in lateral view; *Vm4* at level of *Vl2* in lateral view.

**Chelicerae—**Movable finger sharp and curved distally; serrula composed of 17 hyaline teeth, increasing in size towards distal region, guard tooth rounded. Lamella smooth. Fixed finger with bifid basal tooth, followed by five small teeth decreasing in size (larger tooth subequal to the distal cuspid of the bifid tooth); the last tooth is the biggest, recurved, subequal to the basal cuspid of the bifid tooth, and with an acute apex. Setation: G1 with 3 spatulate setae, first (most dorsal) with peduncle almost smooth; G2 composed of three feathered setae; G3 with 3 setae, the dorsal surface feathered and the ventral serrated; G4 consisting of 3 setae, smooth surfaces, short and thick with thin apex; G5A with six similar sized feathered setae; G5B with 10 setae which are longer than G5A; G6 with one smooth seta longer than half the length of movable finger; G7 with 5 setae decreasing in size from proximal to distal, each feathered from the middle to its apex. Setal group formula: 3-3-3-3-6-10-1-5.

**Pedipalp—**(Fig 4D) All segments without spinose setae. Trochanter: trapezoid in lateral view; with small apical spur present; one ventral row of five large setae with an intermediate row of smaller setae; laterally, frontal projection with a triangular shape with a big setae at the apex; with a mesal row with two setae and one distal spine; this spine can have some small accessory setae. Femur: subcylindrical, two times longer than high, dorsal edge five times longer than ventral edge, thinner at base and wider at apex, two dorsal row of setae, the ectal with six and the mesodistal with two setae; scattered small setae can be found in the distal region; two ventral spines, the proximal bigger; dorsally curved and ventrally “v” shaped. Patella: cylindrical, 2 times longer than high, distal edge 1.3 times longer than basal edge of the segment, with one row of dorsolateral setae, a unique dorso-meso-distal setae, one mesal row of three setae and one ectomesal setae. Tibia: cylindrical, 3 times longer than high, base as high as patella; thinner and longer than patella, with numerous dispersed microsetae, with at least 2 ventral different setae. Tarsus: conical, approximately half the length of tibia, with two dorsolateral rows of setae; one mesoproximal seta; two ventrodistal spines pointing forward; tarsal claw sharp and curved, slightly larger than half tibia length; tarsal spur present.

Unknown female.

**Fig 5:**
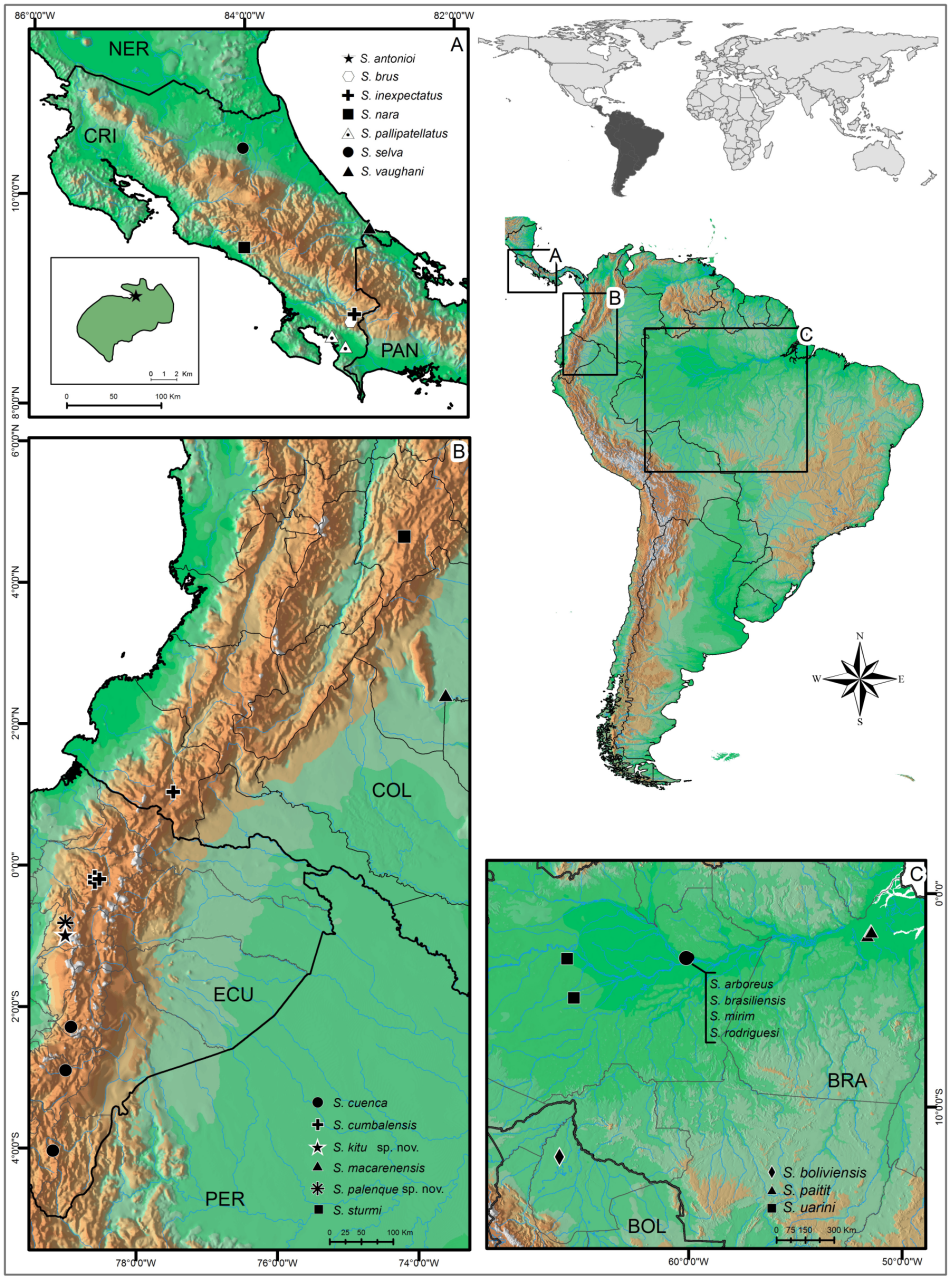
Distributional map of the genus *Surazomus* in Ecuador. (A) Detail of Costa Rica. (B) Detail of part of Ecuador and Colombia. (C) Detail of part of Brazil and Bolivia.

**Distribution—**(Fig 5) Ecuador, Pichincha, Palenque River.

**Remarks—**Armas, Villarreal and Viquez [4] proposed that the white patella I could be a synapomorphy of a group of species composed of *S*. *brus*, *S*. *inexpectatus*, *S*. *nara*, *S*. *selva*, *S*. *pallipatellatus*, *S*. *vaughani*, *S*. *macarenensis* and *S*. *cumbalensis* (the first six from Costa Rica and the last two from Colombia; Fig 5). They argue that, besides the common leg color, all these species have a ventral apophysis in the femur of the pedipalp. *Surazomus kitu* **sp. nov.** has white patella I, so, following Armas, Villarreal and Viquez [4] concept, this species should be included in the *pallipatellatus* species group. However, the new species does not have the ventral apophysis in the pedipalp femur, so this character should not be considered to define the species group since it is not present in all species. Instead, the trilobate male flagellum is in all species with white patella I, so we consider it should be used additionally to diagnose the species group. A phylogenetic analysis of the genus would help understand the distribution and evolution of the characters.

It is worth to note that these characters are present in *S*. *uarini* as well (a species from the Brazilian Amazonia; Fig 5) and was overlooked by Santos and Pinto-da-Rocha [37] in its description. Therefore, *S*. *uarini* should be included in this group.

**Fig 6:**
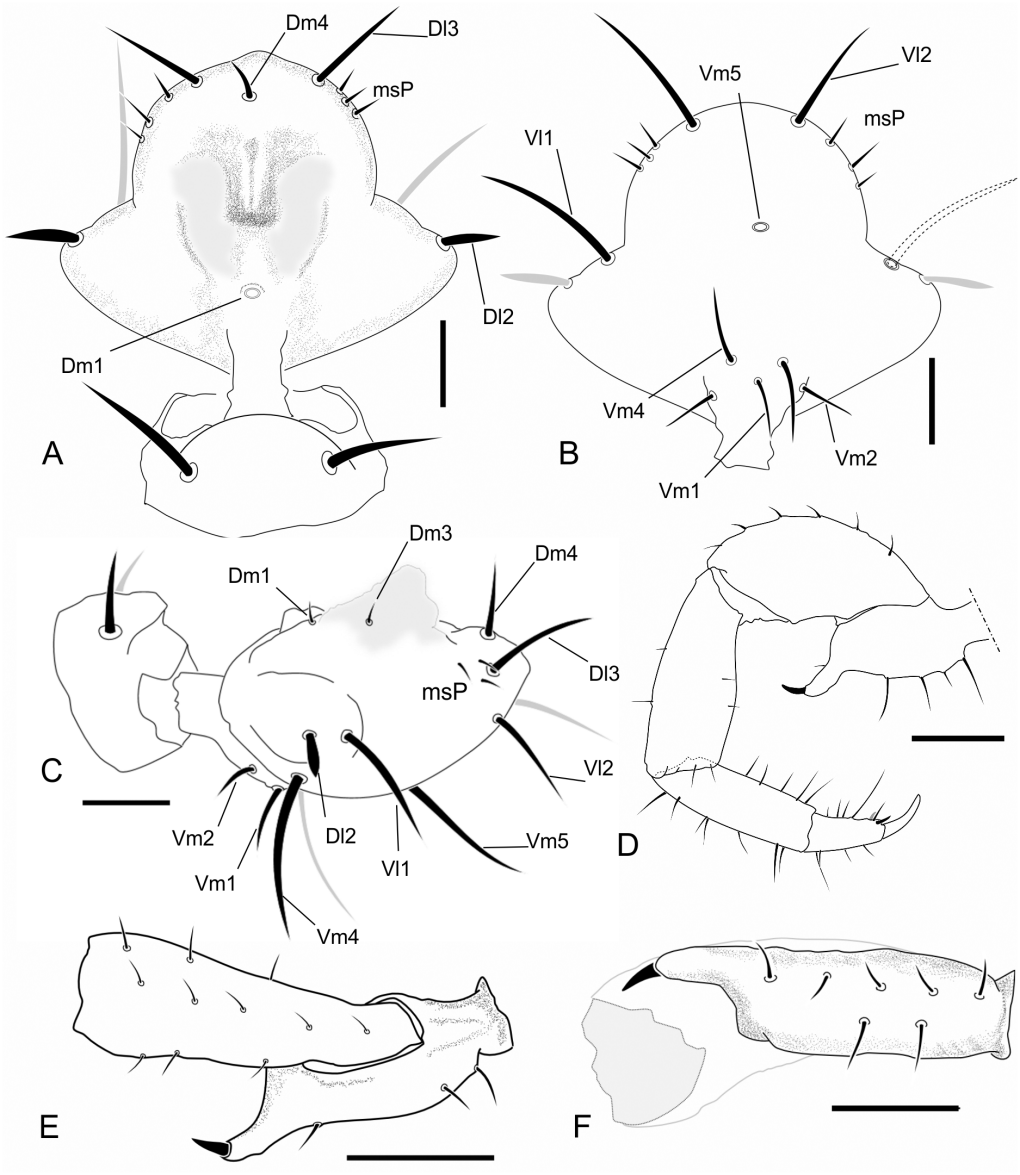
Images of details of the holotype of *Surazomus palenque* sp. n. Dorsal (A), ventral (B), and lateral view (C) of male flagellum. Ectal (D), dorsal (detail of trochanter, E), and ventral view (detail of trochanter, F) of the pedipalp. Scale bar: A: 0.1 mm; B, C, D: 1.0 mm, E-F: 0.25 mm.

**Fig 7:**
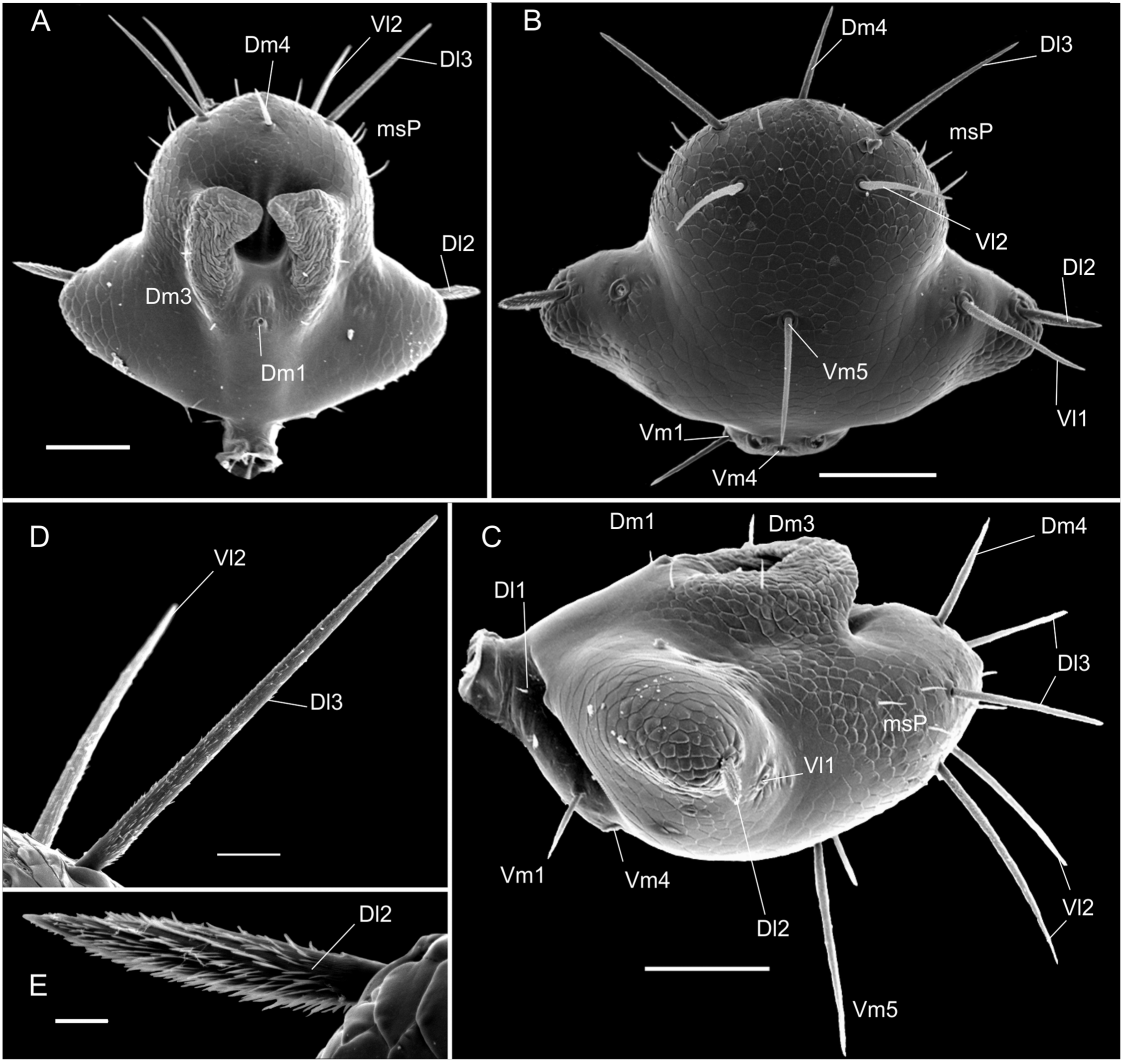
SEM images of details of *Surazomus palenque* sp. n., paratype (MNRJ 04263). Flagellum dorsal (A), ventral (B) and lateral (C) views. Setae *Dl*3 and *Vl*2 (D). Seta *Dl*1 (E). Scale bars: A, B, C: 100 μm; D: 20 μm; E: 10μm.

***Surazomus palenque* sp. nov.**

urn:lsid:zoobank.org:act:FEFD2E5C-44B0-45A4-8578-C3919CA3AB0E

(Figs 3C and 3D; 4; 6A–6F and 7A–7E; Table 3)

**Type material—**Ecuador, Pichincha, C.C. Rio Palenque: holotype (QCAZ): 1 male, palm, 00°54´S 79°00´W, 220m, 27.xii.1980, S. Sandoval; paratypes: (QCAZ) 1 male, palm, 00°54´S 79°00´W, 220m, 21.xii.1980, S. Sandoval; (QCAZ) 1 male, secondary forest, 00°54´S 79°00´W, 220m, 29.xii.1980, S. Sandoval; (MNRJ 04263) 2 males, 00°54´S 79°00´W, 220m, 07.i.1981, S. Sandoval; (QCAZ) 2 males, palm, 00°54´S 79°00´W, 220m, 27.xii.1980, S. Sandoval.

**Diagnosis—**Small species, total length 2.52–2.80 mm (flagellum not included). Male flagellum trilobate with white dorsal spots. Absence of a ventral projection in the pedipalp femur. Trilobate male flagellum, presence of dorsal white spots and absence of ventral spine on the pedipalp femur. Setae *Dl*2 type B. Pedipalps unusually unarmed, without ventral projection on the femur and patella, and patella I totally pigmented; frontal spine on the male pedipalp trochanter strong, curved, and conical with a spiniform setae in its apex, which makes the article look like spiny; lateral and distal lobes of the flagellum in dorsal view fused, in an angle of larger than 90 degrees; anterior projections at each side of the pedicel absent; relative position of *Dl*2 and *V*l1 in lateral view of the flagellum at the same horizontal level. White area of the flagellum very high.

**Etymology—**a noun in apposition referring to the type locality (Palenque River).

**Description—**Male holotype. **Coloration** (Fig 3C and 3D): general pattern light greenish-brown. Chelicerae yellowish and flagellum light brownish. Pedipalps: femur and patella light reddish-brown; tibia yellowish, dark; tarsus reddish-brown. Legs: coxae I–IV, anterior and posterior sterna light yellowish; trochanters I–IV light yellowish-brown; femora I–IV dark greenish-brown; patellae I–IV light greenish-brown; tibiae II–IV light greenish-brown, except for tibia I that is reddish-brown; all tarsus segments slightly clearer. All body setation light reddish-brown.

**Prosoma—**(Fig 3C) Anterior process of propeltidium with 2 setae (one behind the other) followed by 3 pairs of dorsosubmedian setae transversely oriented; eyespot suboval; metapeltidium divided. Anterior sternum with 4+9 setae and posterior sternum with two longitudinal rows of three setae.

**Opisthosoma—**(Fig 3C and 3D) Setae: Tergite II with 3 pairs of anterior microsetae with the posterior pair of setae more distant from each other than the anterior and median pairs, like a trapezium. Segments II–VIII each with 1 pair of large *Dm* setae; segment VIII with small *Dl1*, IX without *Dm*, but pairs *Dl1* and *Dl2* present; *Vm1* and pairs *Vm2*, *Vm3* and *Vl1* present. Segment X same as IX, but without *Dl2*; segment XI same as X, but without *Vl2*. Segment XII same as IX, with *Vm1* and *Vm2* smaller than *Vm3*. Segment XII with a rounded posterodorsal process. Abdominal apodemes with color identical to the rest of the sternites.

**Flagellum—**(Figs 6A–6C and 7A–7E) Flagellum trilobate in dorsal view. With two dorsal and curved white projections bordering the anterior margin of a dorsal depression. With 2 pairs of micro setae at each side of *Dm1* (the proximal being *Dm3A* and the distal one *Dm3B*), and 3 micro setae in the distolateral region. *Dm1* in the anterior third, on a longitudinal elevation, between the bases of the dorsal projections; *Dm4* distal, between *Dl3* in dorsal view. *Dl1* (microsetae) in the middle of the peduncle, proximal to *Vm2*, in the same level as *Dl2*. *Dl2* type B (Fig 6E), on the lateral lobes, slightly posterior to *Dm1* in dorsal view. The remaining setae are type A (Fig 7D). Ventrally convex in lateral view; *Vm2* anterior to *Vm1*; *Vm1* anterior to *Vm4*; the distance between *Vm2* setae larger than that of *Vm4*; *Vm4* larger than *Vm1* and *Vm2*; *Vl1* slightly posterior and more ventral than *Dl2*, at the distal end of the lateral lobe in lateral view; *Vl2* slightly anterior to *Dl3* in lateral view; *Vm4* at the same level of *Vl2* in lateral view.

**Chelicerae—**Movable finger with guard tooth, serrula with 16 hyaline teeth. Fixed finger with bifid basal tooth, followed by five small teeth decreasing in size (the largest tooth subequal to the distal cuspid of the bifid tooth); the last tooth is the bigger, it is recurved, subequal to the basal cuspid of bifid tooth, with an acute apex. Setation: G1 (setae group 1) with 3 spatulate setae, with the peduncle without spicules; G2 composed of 5 subequal feathered setae, longer than the movable finger; G3 with 4 setae, each with surfaces dorsally feathered and ventrally serrated; G4 consisting of 2 smooth, short and thick setae with thin apex; G5 with 8 similar sized feathered setae; G6 with one seta of smooth surface, longer than half the length of the movable finger; G7 with 4 setae decreasing in size from proximal to distal, feathered from the middle to its apex. Setal group formula: 3-5-4-2-8-1-4.

**Pedipalp—**(Fig 6D–6F) All segments without spinose setae. Trochanter: with a trapezoid shape in lateral view, with one ventral row of 4 large setae and frontal projection, digitiform with a pointed seta at the apex, distal spine with small accessory seta at its base. Femur: subcylindrical, 2 times longer than high, dorsal edge 5 times longer than ventral edge, thinner at base and wider at apex, two dorsal rows of setae, the ectal with 7 and the mesodistal with 2 setae; one row of 3 mesal setae, the distal smaller; dorsally curved and ventrally straight (truncated “v”; Fig 5F). Patella: cylindrical, with ventromedial expansion (expansion of the article, from the proximal region to the distal region, with its largest diameter in the median portion; proximal border smaller than the distal), 3.4 times longer than high, distal edge 2.3 times longer than the basal edge of segment, with 3 rows of dorsal setae, 2 ectodistal, 1 ectomedial and 3 ventromesal setae (the last more conspicuous). Tibia: cylindrical, ventrally angled in mesal view (same starting point of the twist of the pedipalp), 3 times longer than high, base thinner than patella, with 2 laterodorsal rows of 4 setae each and two mesodorsal seta, with two mesal rows of setae, the dorsal with 4 and the ventral with 3, some of them plumose, with 4 setae in a curved row. Tarsus: conical, with four plumose setae of which three are arranged in a mesal row; approximately half the length of tibia with numerous setae; tarsal claw sharp and curved, slightly larger than half tibial length; tarsal spur present.

**Distribution—**(Fig 5) Ecuador: Pichincha, Palenque River.

**Fig 8:**
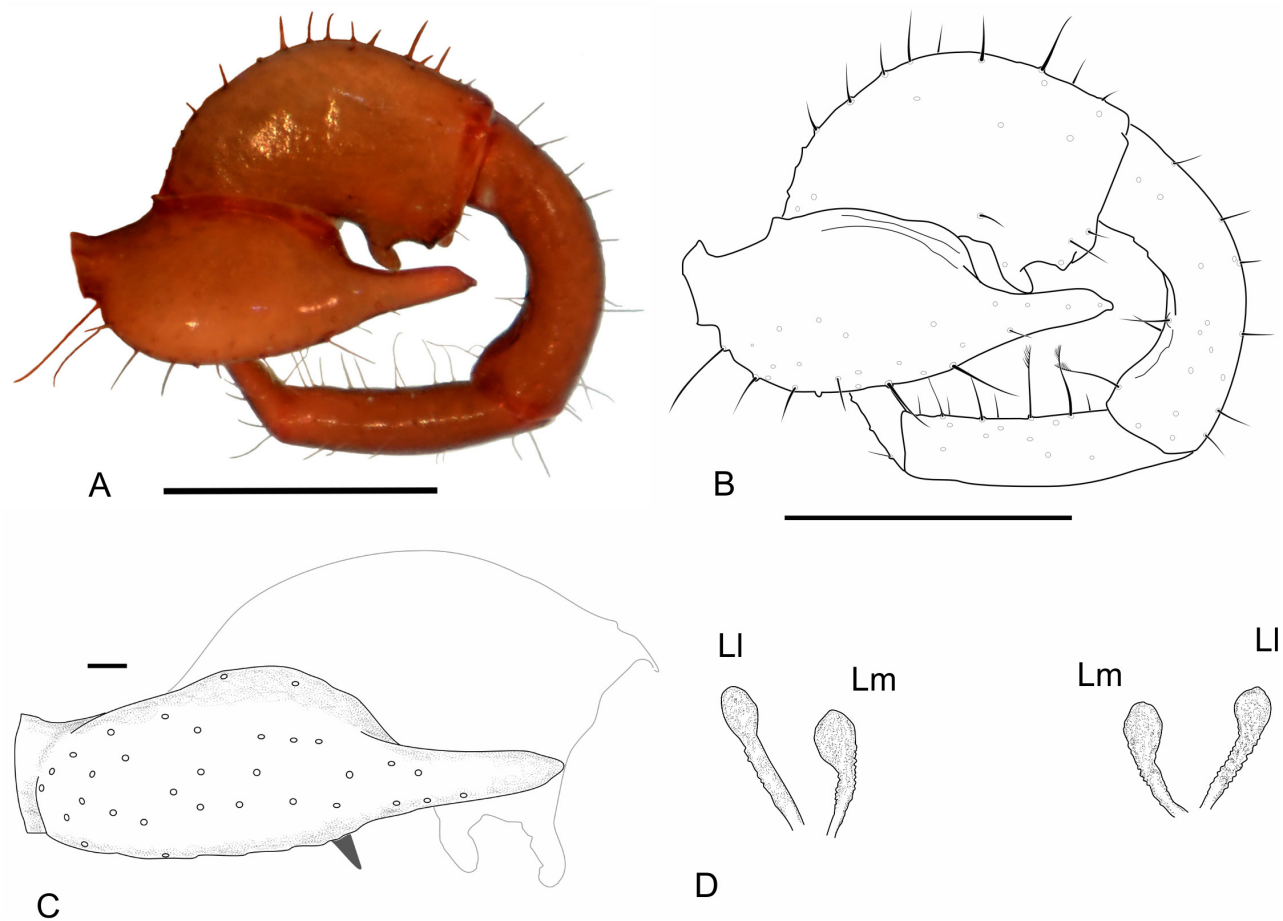
Images of details of *Surazomus cumbalensis* (Kraus, 1957) (MNRJ 04264). Right pedipalp, lateral (A, B) and ventral (C) views. Spermathecae (D). Scale bars: A, B: 1 mm.

**Fig 9:**
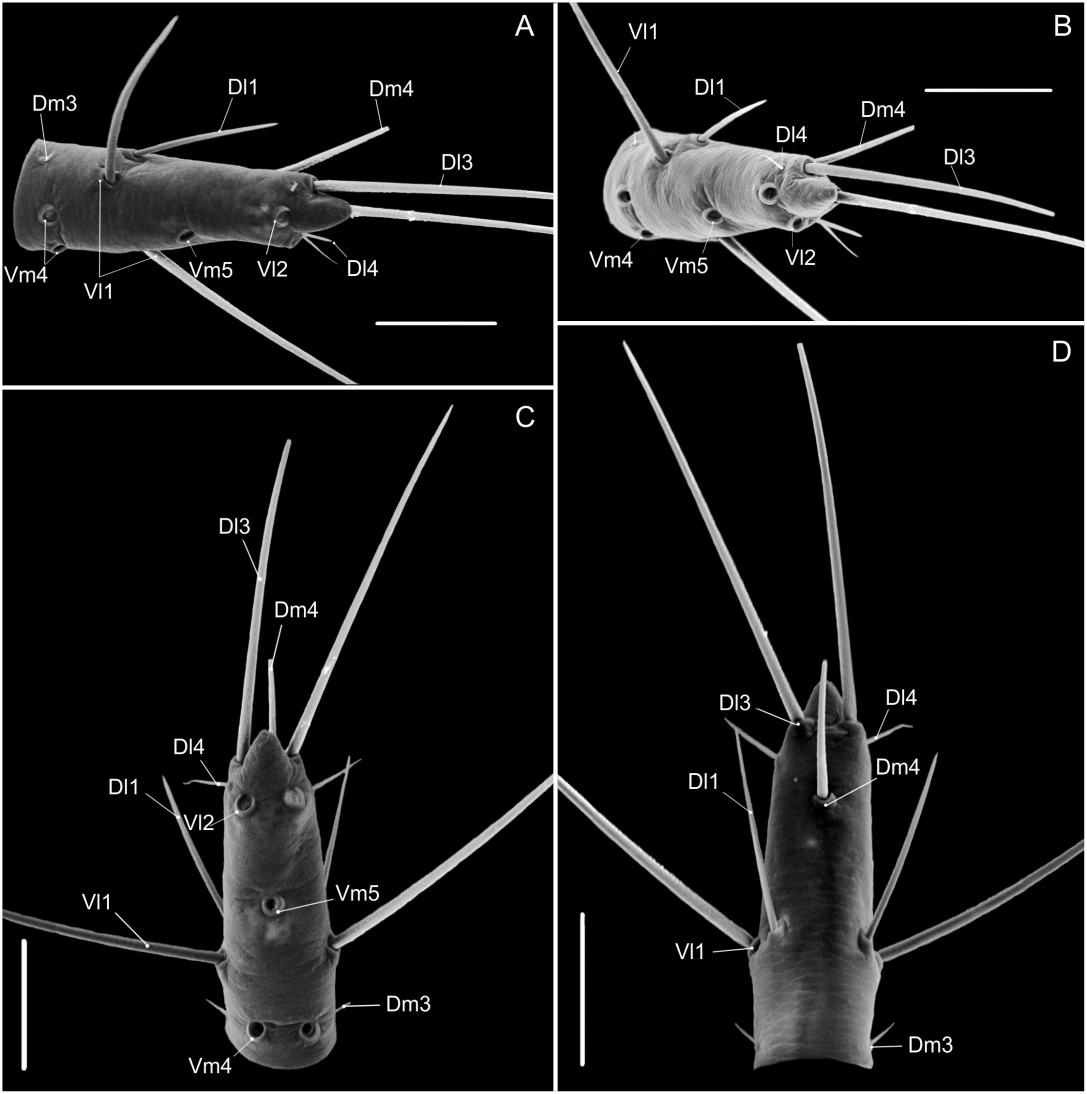
SEM images of the female flagellum of *Surazomus cumbalensis* (Kraus, 1957) (MNRJ 04264). Ventrolateral (A), ventroapical (B) and ventral (D) view. Scale bars, 100 μm.

***Surazomus cumbalensis* (Kraus, 1957)**

urn:lsid:zoobank.org:act:306E5A25-1A95-43AE-BE5C-CDB447C4F59D

(Figs 3G–3H, 5, 8A–8D and 9A–9D)

*Trithyreus cumbalensis* Kraus, 1957: 246–247, Figures 1–6. Rémy, 1961 [40]: 407.

*Schizomus cumbalensis* Rowland and Reddell, 1979 [41]: 162; 1979 [25]: 117, Figures 34, 41, 51, 67.

*Surazomus cumbalensis* Reddell and Cokendolpher, 1995: 119; Reddell and Cokendolpher, 2002: 388; Harvey, 2003: 127; Armas, 2010: 213, Figures d–f.

**Studied material—**(QCAZ) Ecuador, Pichincha, Quito: 1 female, Laderas del Pichincha, 00°10`S 78°30`W, 3000m, 15.i.2000, J.C. Narvaez *leg*; (QCAZ) 1 female, Laderas del Pichincha, 00°10`S 78°30`W, 3000m, 15.i.2000, J.C. Narvaez *leg*; (QCAZ) 1 female, LLOA, 00°14`42”S 78°34`54”W, 3600m, 08.vii.2000, D. Paucar *leg*; (MNRJ 04264) 3 males, 1 female, Laderas del Pichincha, 00°12`S 78°31`W, 28.vii.2000, D. Paucar *leg*.; (QCAZ) 3 males and 1 female, Laderas del Pichincha, 00°12`S 78°31`W, 2900m, 28.vi.2000, D. Paucar *leg*; (QCAZ) 1 specimen (abdomen absent), Pululahua, 16.i.1992, B. Elizalde *leg*.

**Diagnosis—**(complement to the descriptions of Kraus [42] and Rowland and Reddell [41], and the diagnosis of Armas [1])–Large species, total length 4.95 mm (flagellum not included; measurements of the holotype based on Kraus [42]). Presence of two short spiniform projections with a broad base and an acute apex on the posterodorsal border of segment XII, just behind *Dm* seta (Kraus [42], Figure 1). Trochanter with frontal apophysis well-developed, with a rounded tip, without spiniform setae distally. Femur with a bifid ectoventral projection, with rounded apex; the proximal process of the projection curved and larger than the distal. Male flagellum trilobate with posterior lobe cone shaped (in dorsal view) and the angle between the lobes almost reaching 180 degrees, which makes it rounded (in dorsal view), longer than wider. Without white areas dorsally, with a pair of dorsolateral projections in the base of the posterior lobe that surpass the lateroposterior line of the flagellum (in dorsal view). Lateral and median lobes (Ll and Ml, respectively) subequal in length; with distal globose expansion; globose expansion double the width of the stalk. Lateral lobes have the stalk almost two times longer than the distal expansion, while Ml has the stalk and the distal expansion the same length. Ll straight and ML slightly curved. Pairs of lobes distant from each other about five times the width occupied by one pair.

**Male complementary description:**

**Opisthosoma—**(Fig 3G and 3H) Tergite XII without dorsoposterior process, but with two spiniform setae. Setae: Tergite II with three pairs of anterior microsetae with the posterior pair of setae more distant from each other than the anterior and median pairs; sometimes the median setae can be more apart from each other than the other pairs. Sequence of setae on abdominal tergites and sternites, from the medial to the lateral region: Segment V: *Dm*; *As* with 6 setae; *Vm1* positioned anteriorly; an extra pair between *Vm1* and *Vm2*; *Vm2*; an extra pair between *Vm2* and *Vl1*; *Vl1*; *Vl2*. Segment VI-VII: *Dm*; *As* with 6 setae; *Vm* positioned anteriorly; *Vm2*; an extra pair between *Vm2* and *Vl1*; *Vl1*; *Vl2*. Segment VIII: similar to segments VI-VII, however without an extra pair between *Vm2* and *Vl1*; with small *Dl1*. Segment IX: *Dl1*; *Dl2*; *Vm1*; *Vm2*; an extra pair between *Vm2* and *Vl1*; *Vl1*; *Vl2*. Segment X. *Dl1*; *Dl2*; *Vm1*; *Vm2*; *Vl1*. Sternite XII. *Dm*; *Dl1*; *Dl2*; *Vm2*; an extra pair between *Vm2* and *Vl1*; *Vl1*; *Vl2*.

**Flagellum—**Trilobate with posterior lobe cone shaped (in dorsal view) and the angle between the lobes almost reaching 180 degrees, which makes it rhombus (in dorsal view), longer than wider. Without white areas dorsally, with a pair of dorsolateral projections in the base of the posterior lobe that surpass the lateroposterior line of the flagellum (in dorsal view). *Dl1* slightly anterior to *Vl1* in lateral view. The region between *Dl1* and the lateral projections is the highest of the flagellum, in lateral view.

**Chelicerae—**Movable finger without guard tooth, serrula with 16 hyaline teeth. Fixed finger with bifid basal tooth, followed by five small subequal teeth. Setation: G1 (setae group 1) with 3 spatulate setae, all with basal surface almost smooth; G2 composed of 5 feathered setae, all subequal, longer than the movable finger; G3 with 3 setae, each consisting of dorsally feathered and ventrally serrated surfaces; G4 consisting of 3 smooth, short and thick setae, with thin apex; G5 with 20 similar sized feathered setae; G6 with one smooth setae, longer than half of movable finger length; G7 with 7 setae decreasing in size from proximal to distal, feathered from the middle to its apex. Setal group formula: 3-5-3-3-20-1-6.

**Pedipalp—**(Fig 8A–8C) Trochanter with frontal apophysis well developed, with a rounded tip, without spiniform setae distally. Femur with a bifid ectoventral projection, with rounded apex; proximal process of the projection curved and longer than the distal process. Patella strongly curved ventrally.

**Female complementary description:**

**Flagellum—**(Fig 9A–9D) With 3 segments, approximately 3–4 times longer than wide. **Segment I** lost and not observed. **Segment II** lost and not observed. **Segment III-IV** with *Dm3* (microsetae pair), *Dm4*, *Dl1* (pair), *Dl3* (very large pair), *Vm4* (very large pair); *Vm*5, *Vl1* (very large pair), *Vl2* (very large pair). *Dl4* positioned between *Dl3* and *Vl2*; *Vl1* located proximal to *Dl1; Dm3* more proximal than *Vm4*, *Dl3* clearly more distal than *Vl2*. *Dm4* closer to *Dl3* than to *Dl1*.

**Spermathecae—**(Fig 8D) With lateral and median lobes (Ll and Ml, respectively) subequal in length; with distal globose expansion; globose expansions double the width of the stalk. Lateral lobes have the stalk almost two times longer than the distal expansion, while the Ml has the stalk and the distal expansion with the same length. The Ll is straight and the Ml slightly curved (Fig 7D).

**Distribution—**(Fig 5) Colombia (Nariño) and Ecuador (Pichincha).

**Fig 10:**
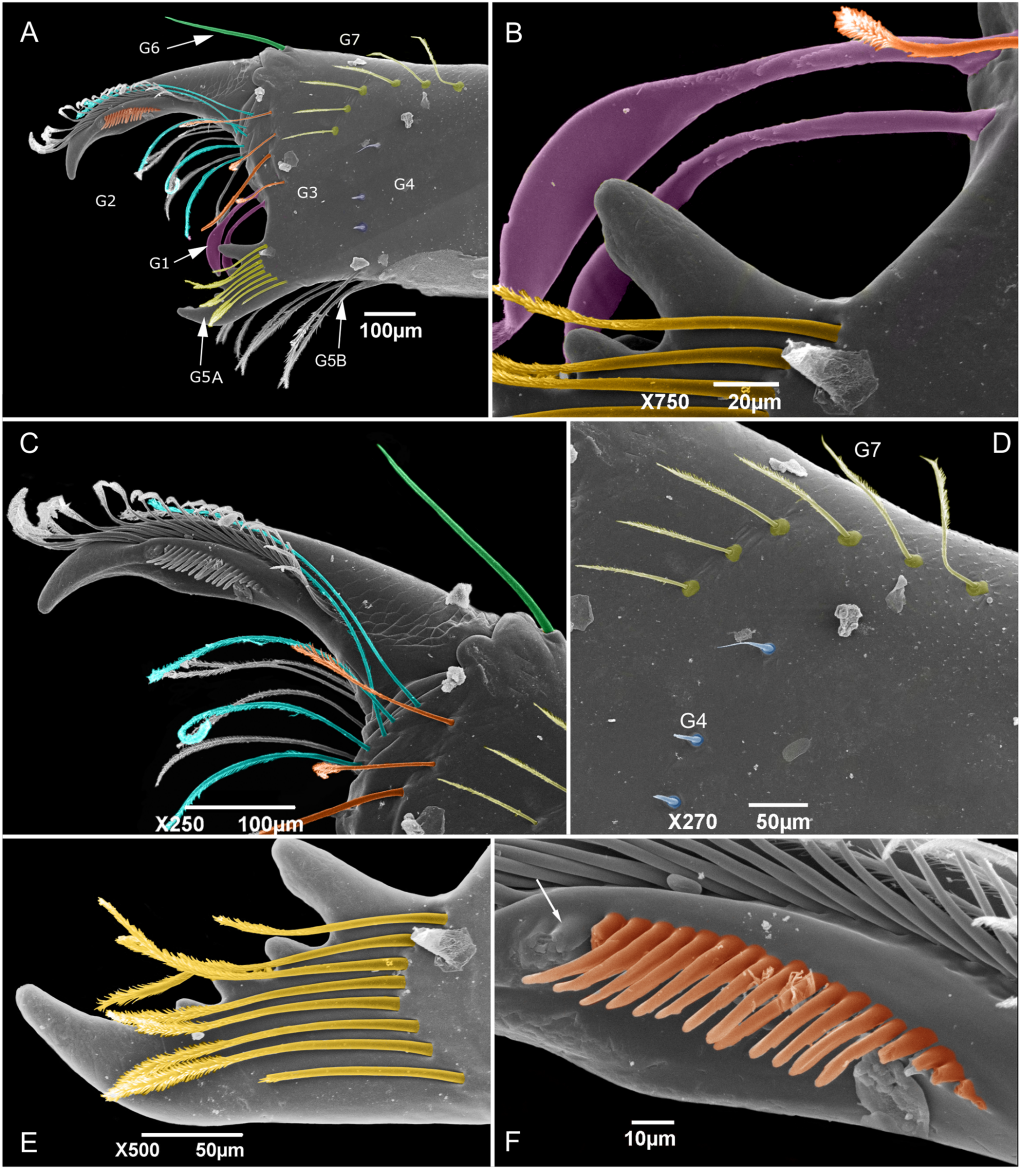
SEM images of details of the chelicerae of *Surazomus cuenca*, female. (A) Right chelicera, mesal view showing all groups of setae. (B) Setae G1. (C) Detail of movable tooth. (D) Setae G7. (E) Fixed tooth and detail of distal portion of setae G5. (F) Detail of the serrula. Scale in the images.

**Fig 11:**
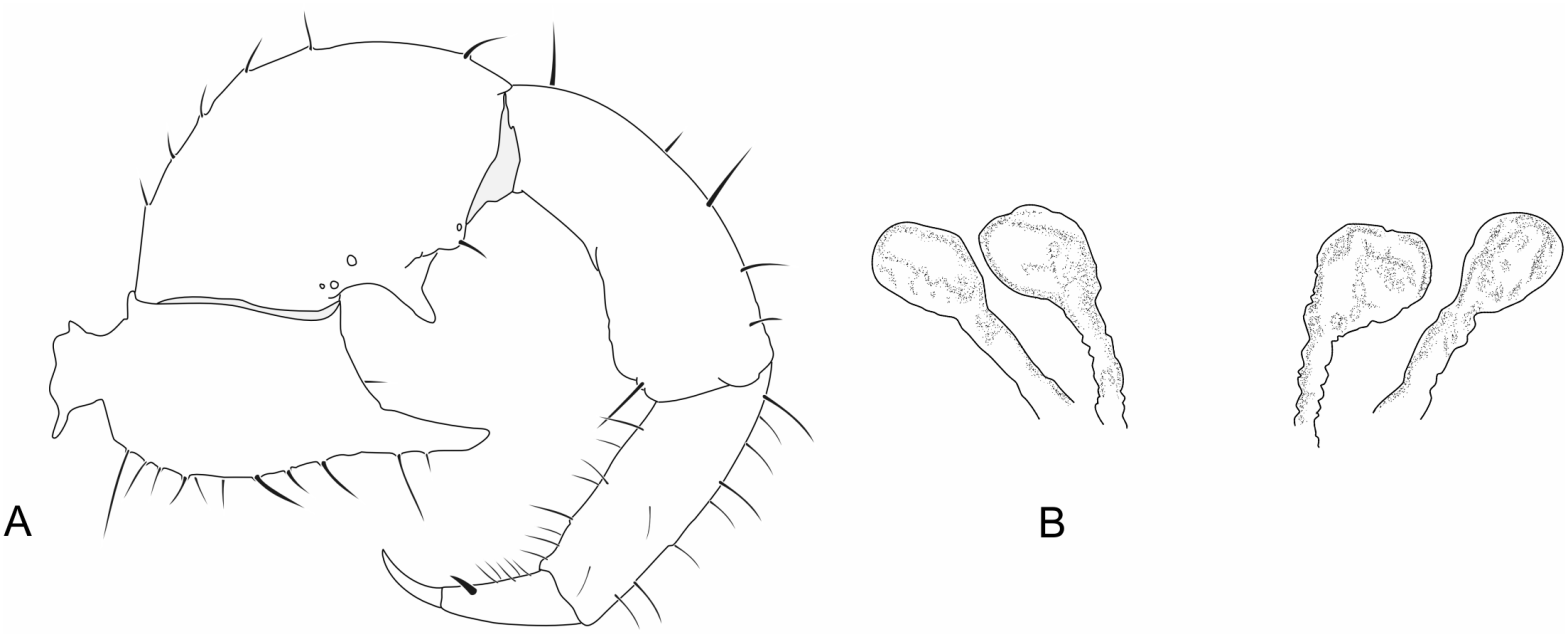
Images of details of *Surazomus cuenca* (Rowland and Reddell, 1979) (MNRJ 04265). (A) Male pedipalp, ectal view. (B) Spermathecae, dorsal view. Pedipalp scale bars: 1 mm.

**Fig 12:**
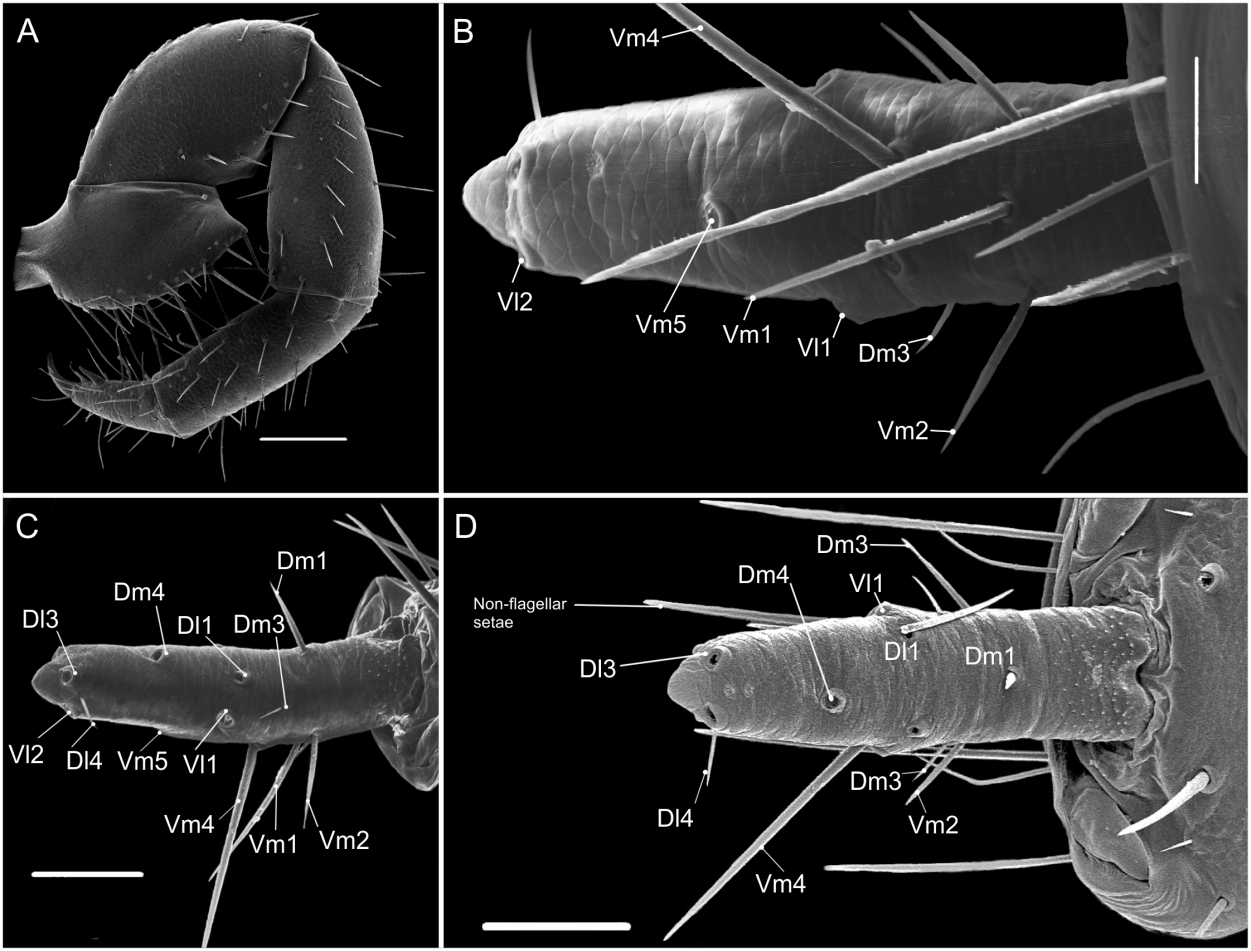
SEM images of details of *Surazomus cuenca* (Rowland and Reddell, 1979) (MNRJ 04265). (A) Female pedipalp, ectal view. (B-D) Female flagellum. (B) Ventral view. (C) Lateral view. (D) Dorsal view. Scale bars: A. 1 mm; B. 50 μm. C-D. 100 μm.

***Surazomus cuenca* (Rowland and Reddell, 1979)**

urn:lsid:zoobank.org:act:091BC872-06D3-4B52-985A-02096BBB0169

(Figs 3E–3F, 5, 10A–10F, 11A–11B and 12A–12D; Table 3)

*Schizomus cuenca* Rowland and Reddell, 1979 [25]: 107–109, Figures 34, 40, 50, 53, 58.

*Surazomus cuenca* (Rowland and Reddell): Reddell and Cokendolpher, 1995: 118–119; Reddell and Cokendolpher, 2002: 388; Harvey, 2003: 127; Armas, 2010: 213, Figures 14a–c.

**Studied material—**Holotype, *Schizomus cuenca*, AMNH: Ecuador, Cuenca, D02I, 3.iv.1942, HEF and DLF leg. (MNRJ 04265), 2 males and 8 females, Ecuador: Loja, Zamora Huayco, Private Reserve El Madrigal, 4° 2'23.17"S, 79°10'30.53"O, alt. 2200m, 27.iii.2011; A. Chagas, A. Giupponi, and A. Kury *leg*. Chunchi. 8.x.1995 G. Onore *leg*. 1 female.

**Diagnosis—**Total length 4.00–4.33 mm (flagellum not included). Setae of chelicerae: G4 with three setae; G5 divided in G5A and G5B; not present in *Rowlandius* sp. and *Piaroa* sp.). Trochanter with frontal apophysis well developed and without distal spiniform setae. Femur with ectoventral projection short and rounded, the proximal projection large and acuminated. Dorsoventrally flattened, sub-rhomboidal, trilobate and short (as long as wide). Spermathecae with lateral (Ll) and median lobes (Ml) subequal in length, with an apical globose expansion; globose expansion three to four times the width of the stalk. Stalk about the same length as the expanded region. Pairs of lobes distant from each other about one times the width occupied by one pair.

**Male complementary description** (based on the recently collected material; variation in parenthesis):

**Prosoma—**(Fig 3E) Anterior process of propeltidium with two setae (one behind the other) followed by three pairs of dorsosubmedian setae; eyespot sub-oval; metapeltidium divided. Anterior sternum with 2+14 setae and posterior sternum with 6 setae.

**Opisthosoma—**(Fig 3E and 3F) Posterodorsal process of segment XII short and broad (forming a lamina with convex border with slightly conical apex), not surpassing 1/3 the length of the *Dm* setae (see Rowland and Reddell [25], Figure 58). **Setae**: Tergite II with 3 pairs of microsetae. Segment V: *Dm*; *Vm1*; one supernumerary pair between *Vm1* and *Vm2*; *Vm2*; one supernumerary pair between *Vm2* and *Vl1*; *Vl1* and *Vl2*. Segment VI: *Dm*; *As* with 3–4 setae; *Vm2*; *Vl1*; *Vl2*. Segment VII. *Dm*; *As* with 3 pairs of setae; *Vm1* anteriorly; with pairs *Vm2*; *Vl1* and *Vl2*. Segment VIII: *Dm*; *Dl2*; *As* with 3 pairs of setae; with *Vm2*, *Vl1* and *Vl2* pairs. Segment IX: with *Dl1* and *Dl2; Vm1*; *Vm2*; one supernumerary pair between *Vm2* e *Vl1*; *Vl2*. Segment X: with *Vl1* and *Vl2*; *Vm1*, and pairs *Vm2*, *Vl1* and *Vl2*. Segment XI: with pairs *Dl1*, *Vm2* and *Vl1*. Segment XII: *Dm1*, *Dl1* and *Dl2*; with pairs *Vm2*, *Vl1* and *Vl2* and a supernumerary pair between *Vm2* and *Vl1*. Segment XII: with the dorsomedial part laminar shape, slightly larger than the posterior border, with a pair of setae in the lateral distal apex. Abdominal apodemes narrow and oval, slightly sclerotized and darker than sternites. **Flagellum**, male. Dorsoventrally flattened, sub-lozenge shaped, trilobate and short (as long as wide). **Setation**: *Dm1* in lateral view just before (in the base of) the median elevation of the flagellum; *Dm1* more proximal than *Dl1*; *Dl1* slightly proximal than *Vl1*. *Dl3* slightly anterior to *Dm4* and *Vl2*. Dorsomedial depression of flagellum "W"-shaped in dorsal view (see Rowland and Reddell [41], Figure 40), without a "bridge" linking the anterior and posterior elevations; the tips of the depression are not linked to each other posteriorly, forming the apex of the “W” (the tips are divergent, not connected). With a group of laterodistal microsetae between *Dl2* and *Dl3*.

**Chelicerae—**(Fig 10A–10F) Movable finger (Fig 10A and 10C) sharp and curved distally, serrula, composed of 19 hyaline teeth, increasing in size towards distal region, guard tooth large. Lamella simple. Fixed finger (Fig 10A and 10E) with 3 similar sized teeth between two larger outer teeth. Setation: G1 (setae group 1) with 3 spatulate setae with smooth stalk surface; G2 composed of 6 feathered setae; G3 with 4 setae, each consisting of dorsal feathered and ventral serrated surfaces; G4 consisting of 3 smooth, short and thick setaewith thin apex; G5A with 8 similar sized feathered setae; G5B with 8 setae, G6 with one smooth setae longer than half of movable finger length. Setal group formula: 3-6-4-3-8-8-1.

**Pedipalp—**(Fig 11A) All segments smooth, without spinose setae. Trochanter: with mesal spur, with two ventral rows of setae; setae of the mesal row bigger than those of the ectal row; with frontal apophysis well developed, without distal spiniform seta (this seta is present in the holotype). Femur: 1.9 times longer than high, dorsal edge 3.5 times longer than the ventral edge; lateral external surface with 4 setae. With ectoventral projection short and rounded, the proximal projection large and acuminated. Patella: strongly curved ventrally and slightly thickened in the distal portion, three times longer than high. Tibia: cylindrical, 3.3 times longer than high. Tarsus: conical, approximately 2.2 times the tibial length. Tarsal claw sharp and curved, about 0.25 times the tibial length. Tarsal spur present.

**Female complementary description:**

**Flagellum—**(Fig 12B–12D) With 3 segments, approximately 3–4 times longer than wide. **Segment I** with no setae, with only tiny triangular spines, similar to bristly scales, mainly in the dorso-lateral portion. **Segment II** with *Dm1*, *Vm1* and *Vm2* (pair). **Segment III IV** with *Dm3* (microsetae pair), *Dm4*, *Dl1* (pair), *Dl3* (very large pair), *Vm4* (very large pair); *Vm5*, *Vl1* (very large pair), *Vl2*. *Dl4* between *Dl3* and *Vl2*; *Dl1* more proximal than *Vl1*; *Dm3* more proximal than *Vm4; Dl3* and *Vl2* nearly forming a vertical line. *Dm4* slightly closer to *Dl1* than to *Dl3*.

**Pedipalp—**(Fig 12A) Similar to male, but without frontal projection on the trochanter or ventral projections on the femur. Patella not curved ventrally, in lateral view.

**Spermathecae—**(Fig 11B) With lateral (Ll) and median lobes (Ml) subequal in length, with an apical globose expansion; globose expansion three to four tumes the width of the stalk. The stalk about the same length as the expanded region.

**Distribution—**(Fig 5) Cu enca and Zamora, Azuay province, Ecuador.

## Discussion

### The cheliceral setae in Hubbardiidae and Protoschizomidae

The cheliceral setae of Hubbardiidae were described by Lawrence [8], who detailed their shape, texture, size and position in an unidentified species of the genus “*Schizomus”*. Lawrence’s definition was and still is followed by several authors describing Hubbardiidae species. In Protoschizomidae, the first detailed description of the cheliceral setae was published by Cokendolpher and Reddell [13]. Besides that, some figures of other Protoschizomidae are available in the literature [13, 16, 28], and on the Internet (*Agastoschizomus lucifer*; [30]). The two schizomid families show clear differences in number and position of the setae; for example, Protoschizomidae have fewer teeth on the fixed finger, serrula with rounded knobs, and setae G5A (the cheliceral brush of Cokendolpher and Reddell [13]), absent (in general, Protoschizomidae have less setae on the chelicerae than its sister group, the Hubbardiidae (Fig 2)).

The groups of setae have been frequently used by different authors, but confusion has been made with some of them, such as the G4 (see below). To avoid this, we present a new definition to the known groups and propose new ones, as follows:

- **Group 1 (G1):** alternatively named as blood hairs by Hansen and Sorensen [7], it was defined by Lawrence [8] to include three setae located dorsally to the fixed teeth. These three setae are found in both schizomid families, but their shape may vary considerably, as described in Lawrence [8]: in Hubbardiidae, the setae of G1 have the sub distal portion clearly swollen, sometimes with spicules on the stalk or on the blade; in Protoschizomidae they are long and cylindrical, similar to most of the other cheliceral setae.
- **Group 2 (G2):** this group is located on the membranous area, close to the insertion of the movable finger, and dorsally to G1. In Hubbardiidae G2 has lots of setae (from three, in *Stenochrus mexicanus* (Rowland, 1971), to nine, in *Piaroa pioi* Villarreal, Armas and García, 2014), while in Protoschizomidae it is composed by one (in most of the species where this setae is known) or six long and thin setae (this is known only in *Agastoschizomus lucifer*).
- **Group 3 (G3):** in Hubbardiidae these setae are located close to the membranous area in small number, and are thin; in Protoschizomidae, they form a row on the frontal border of the chelicerae and are thick.
- **Group 4 (G4):** Lawrence [8] described G4 as a group with 2 to 5 setae positioned in the mesal side of the chelicerae. In his illustration, Lawrence [8] includes 3 setae in the group, but we consider his G4 as encompassing two groups, G4 and G7 (the last one, a new group defined here); Lawrence’s [8] dorsalmost setae (the larger and more apart from the two ventral setae) is part of the new setae group G7, and the two small ventral setae are part of G4. This misunderstanding of G4 and G7 is also found in other publications in which G4 includes both groups. For example, in the description of *S*. *rodriguesi*, Cokendolpher and Reddel [24] states "[…] group 4 = 5 long dorsally, 2 short ventrally…"; the “5 long dorsally” is the current G7 and the “2 short ventrally” is the current G4. This was made with several *Surazomus* species, such as *S*. *arboreus*, *S*. *boliviensis*, *S*. *brasiliensis*, *S*. *cuenca*, *S*. *manaus*, *S*. *mirim*, *S*. *paitit* and the aforementioned *S*. *rodriguesi*. The only species with G4 correctly described is *S*. *uarini* [37]. All other species of the genus never had their cheliceral setae described. In general in Hubbardiinae, G4 has only two short and stout setae positioned on the middle of the mesal side of the chelicera (e.g. *Piaroa*, [18], Figure 26; *Wayuuzomus gonzalezspongai*, Fig 2B). Regarding Megaschizominae, the only species with its chelicerae known is *Megaschizomus mossambicus* and it has three long and thick setae with similar shape and placement as those of Hubbardiinae. The family Protoschizomidae lacks the G4 group of setae (Fig 2A). Therefore, G4 seems to be a putative synapomorphy of Hubbardiidae, composed by two to four short and stout setae, but only a cladistic analysis can actually show the character support of the groups. In *Surazomus*, from the total of eleven species in which the number of setae on the chelicerae is described, one has 4 setae (*S*. *boliviensis*), eight have 3 setae (*S*. *cumbalensis*, *S*. *cuenca*, *S*. *brasiliensis*, *S*. *arboreus*, *S*. *manaus*, *S*. *mirim*, *S*. *kitu* **sp. nov.** and *S*. *palenque* **sp. nov.**), and two have 2 setae (*S*. *rodriguesi*, *S*. *uarini*).
- **Group 5 (G5):** the concept of this group is broadened to include all setae in the ventral face of the chelicerae. All these setae have a plumose apex, but, as they have different sizes,positions, and shape of the cuticle where they are inserted, they are separated in subgroups, G5A and G5B. G5A is equivalent to the G5 *sensu* Lawrence [8] and subsequent authors (e.g. [18], pg. 374). All setae of this subgroup are in the base of the fixed tooth; besides this, the setal insertions of G5A do not have a prominent border, as seen in the base of setae G5B (Fig 10A, 10B and 10E). G5A and G5B can form a single row of setae, as in *Surazomus kitu* **sp. nov.**, or may form separate rows, as in *S*. *cuenca* **sp. nov**.
- **Group 6 (G6):** this group consists of only one long and conspicuous seta. In Protoschizomidae it is positioned slightly mesal (Fig 2A), and in Hubbardiidae it is located dorsomesally (Figs 2B, 10A and 10C).
- **Group 7 (G7):** studying different genera of Hubbardiinae (*Piaroa*, *Stenochrus*, *Surazomus* and *Wuayuuzomus*) we noticed the repeated occurrence of a dorsomesal row of cylindrical setae forming a curved line, starting proximally more dorsal, and ending distally near the medial line of the chelicerae. We propose that these setae form a group here called G7, since they are not clearly assignable to any of Lawrence’s groups. In Protoschizomidae this group has only two long and sharp setae, positioned on the middle area of the chelicerae (Fig 2A), while in Hubbardiinae, 5–7 aligned setae can be found (Figs 2B, 10A and 10D).

**Serrula—**In Protoschizomidae the serrula teeth are short and few in number (e.g., eight in *Protoschizomus franckei*). In Hubbardiidae the serrula teeth are long, resembling a seta (e.g. *Surazomus cuenca*, Fig 10A and 10F; *Piaroa pioi* Villarreal *et al*., 2014, Figures 32, 35, 36; *Piaroa guipongai* Villarreal *et al*., 2012), with much higher number of teeth (around 18).

**Teeth on the fixed finger—**The number of teeth on the fixed finger of Protoschizomidae is the same as the Schizomida sister group Thelyphonida [38]; both have two teeth, but thelyphonids have many more setae in the movable finger and in the ventral and frontal region of the basal segment (Haupt [43], Figures 1, 2) than Schizomida. This possible plesiomorphic character present in Schizomida must occur due to common ancestry, since these groups form the long recognised clade Uropygi [44–54].

The setae and teeth of the chelicerae, therefore, provide a number of characters to differentiate species, genera, families, and will also be useful for future phylogenetic analyses. The number, size, position and texture of the setae (as discovered by Lawrence [8]) have useful information when their correct homology is known, as proposed in the present paper. For example, among the seven groups of setae on the mesal side of the chelicerae, taxonomic implications can be observed at the specific, generic and familial level with G4 and G5 (the first group is present (as far as known) only in Hubbardiidae, and the second is present as divided in G5A and G5B only in Hubbardiinae—G5B absent in Megaschizominae). The equivalent of all these setae in other orders (such as Thelyphonida) is not known, so statements about transformations of characters are premature and would be speculative. On the other hand, the number of teeth in the fixed finger shows similarities between Protoschizomidae and Thelyphonida (as stated above) suggesting the presence of plesiomorphic character states in Protoschizomidae; so, the different character states present in Hubbardiidae (e.g. the presence of G4) could be synapomorphies of the group.

### Chaetotaxy of the male flagellum

Villarreal *et al* [18] called attention to one pair of dorsobasal microsetae on the male flagellum of several Hubbardiinae species, and proposed its homology with the *Dm3* of Protoschizomidae. They also commented that this pair of setae must be more common than registered in the literature. Accordingly, all species of *Surazomus* here studied have *Dm3*. In addition to this, another pair was observed when analyzing the flagellum of *S*. *palenque* **sp. nov.** (Fig 7A and 7C); this extra pair of microsetae is located in the middle of the dorsal projection of the flagellum. To maintain stability of the traditional chaetotaxy in the literature, we name this second pair as *Dm3B* and the original *Dm3* as *Dm3A*. The new pair of setae (*Dm3B*) is also present in *Surazomus uarini* (see Santos and Pinto-da-Rocha [37], Figure 7). We highlight the presence of these setae as they can play important roles in the understanding of the taxonomy and systematic of Schizomida, in combination with other characters.

### Number of segments on the female flagellum

The females of Schizomida have in general three to six flagellum segments. The Protoschizomidae, which are considered to have mostly primitive character states [13], has flagellum with three to six segments, depending on the genera and species. The Hubbardiidae, which is considered as possessing derived character states, has females with three or four segments (Hubbardiinae) or six segments (Megaschizominae) [13]. Cokendolpher and Reddell [13] stated that the number of segments in Hubbardiinae, specifically the three-segmented flagellum, is the plesiomorphic state, as it is widespread in different groups in the subfamily, but this does not seem to be the most parsimonious interpretation, since a widespread character in a population or in a genus does not necessitate plesiomorphy; this interpretation should be evaluated under a phylogenetic context, which is not addressed here.

**Fig 13:**
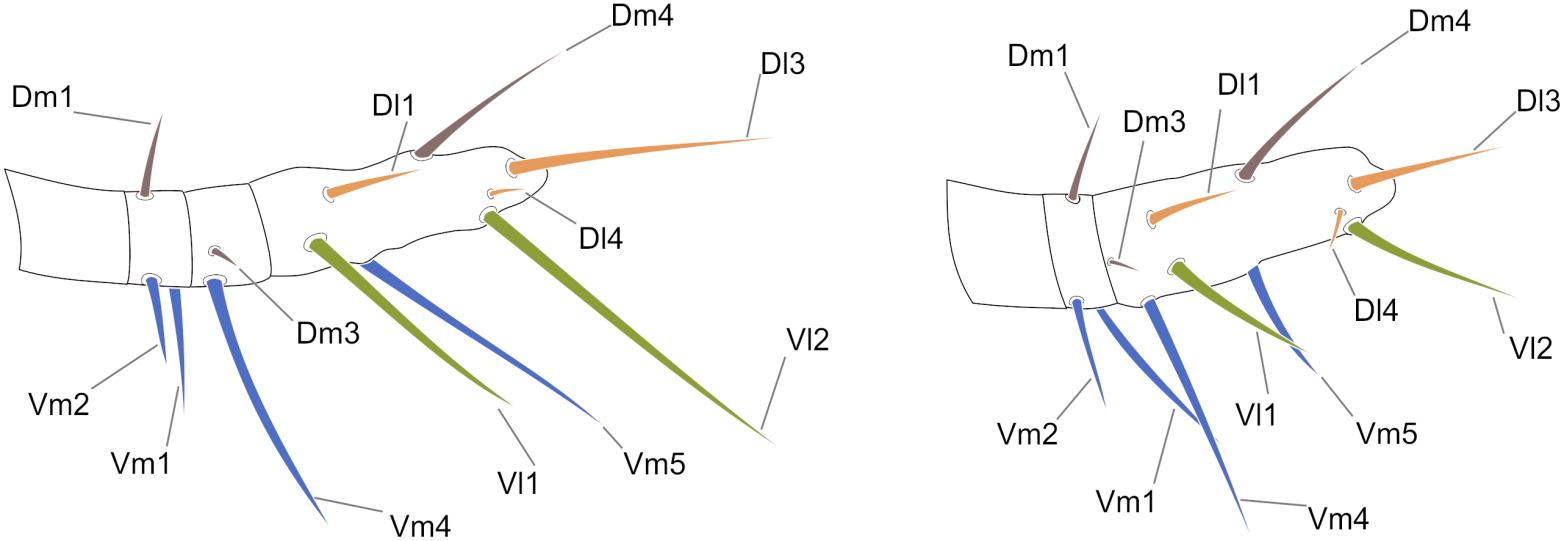
Female flagellum of a Protoschizomidae and of a Hubbardiidae (Hubbardiinae) in lateral view to show the fusion/split shown by the position of the setae. The colors represent the homologous setae in both flagella.

The lower number of segments in the females of Hubbardiinae can be considered as the result of two factors: 1) ‘presence of three segments’ is the primitive character state and did not change over time (as in part of the Protoschizomidae, even though this event has not been tested); 2) ‘presence of three segments’ is a reduced-number-flagellum as the result of loss or fusion of one or more segments from an ancestral taxon with more segments (Fig 13). The homology of these segments beyond Hubbardiinae genera and species is out of the scope of this article.

Studying the flagellum of the species here presented and those available on the literature, we concluded that, at least in *Surazomus*, segment III in trimerous flagella is homologous to segments III and IV in tetramerous flagella based on the setal distribution. At the present state of knowledge, it is not possible to define whether flagella with three segments are the result of fusion of segments III and IV or the augmented number of segments results from a subdivision event of tri-segmented flagella. The distribution pattern of setae in the last segment of three-segmented species is identical to that found in segments III and IV of *Calima*, *Mayazomus* and *Piaroa* (Fig 13) [14, 18, 27, 35]. Consequently, the identification of the flagellar segments in three segmented species of Hubbardiinae should be (from proximal to distal): segment I, segment II and segment III-IV.

An interesting feature is that the female flagellum of *Surazomus* is smaller than that of other genera with higher numbers of segments in Hubbardiinae (e.g. *Piaroa*). Even if it is not known whether a lower number of segments results from fusion or subdivision of segments, the reduction in segment numbers can be related to a shortening of the flagellum. This is evident by the position of *Vm5* in relation to *Vm4* and *Vl2* which is very far in *Piaroa* and close in *Surazomus*; the same happens with *Dm3* in relation to *Dl1* and *Vl1*, the first seta is distant from the pair in *Piaroa* and close in *Surazomus*.

### Species groups and relationship

At least two species groups can be detected within *Surazomus*. One is composed by *S*. *arboreus*, *S*. *manaus*, and *S*. *paitit* (all Amazonian) and is diagnosed by (in males) the shape of the flagellum, the presence of two weak dorsomedian pits on the flagellum (one behind the other in a longitudinal row), by a ventrodistal expansion on the pedipalp femur, and by the presence of a single dorsoposterior and large projection on tergite XII. Additionally, the female spermathecae have the genital lobes ending in a small projection.

The second group comprises *S*. *brus*, *S*. *cuenca*, *S*. *inexpectatus*, *S*. *kitu* **sp. nov**., *S*. *nara*, *S*. *palenque* **sp. nov.**, *S*. *selva*, and *S*. *vaughani* (from Costa Rica, Colombia and Ecuador). It has more diverse male flagellar shapes than the other group, and is defined by males with tergite XII without a single dorsomedian digitiform projection and presence of white and membranous areas on the dorsal surface of the flagellum. The two new species here described belong to this group, but unlike *S*. *brus*, *S*. *inexpectabilis*, *S*. *selva*, *S*. *nara* and *S*. *vaughani* (all from Costa Rica), they lack the two conical dorsal tubercles on tergite XII and they do not have the distal projection (spine) on the patella of the pedipalps.

All other known species of *Surazomus* are not assigned to any group yet. A systematic review is needed to confirm the relationship of the species of this genus, and is already being carried out (Villarreal *et al*., in prep.).
